# *In Vitro* Micropatterned Human Pluripotent Stem Cell Test (µP-hPST) for Morphometric-Based Teratogen Screening

**DOI:** 10.1038/s41598-017-09178-1

**Published:** 2017-08-17

**Authors:** Jiangwa Xing, Yue Cao, Yang Yu, Huan Li, Ziwei Song, Hanry Yu

**Affiliations:** 1 Institute of Bioengineering and Nanotechnology, A*STAR, The Nanos, #04-01, 31 Biopolis Way, Singapore, 138669 Singapore; 2 Mechanobiology Institute, National University of Singapore, T-Lab, #05-01, 5A Engineering Drive 1, Singapore, 117411 Singapore; 3 BioSyM, Singapore-MIT Alliance for Research and Technology, Enterprise Wing 04-13/14 and B1, 1 Create Way, Singapore, 138602 Singapore; 4 Department of Physiology, Yong Loo Lin School of Medicine, MD9-04-11, 2 Medical Drive, Singapore, 117597 Singapore; 5 Gastroenterology Department, Southern Medical University, Guangzhou, 510515 China

## Abstract

Exposure to teratogenic chemicals during pregnancy may cause severe birth defects. Due to high inter-species variation of drug responses as well as financial and ethical burdens, despite the widely use of *in vivo* animal tests, it’s crucial to develop highly predictive human pluripotent stem cell (hPSC)-based *in vitro* assays to identify potential teratogens. Previously we have shown that the morphological disruption of mesoendoderm patterns formed by geometrically-confined cell differentiation and migration using hPSCs could potentially serve as a sensitive morphological marker in teratogen detection. Here, a micropatterned human pluripotent stem cell test (µP-hPST) assay was developed using 30 pharmaceutical compounds. A simplified morphometric readout was developed to quantify the mesoendoderm pattern changes and a two-step classification rule was generated to identify teratogens. The optimized µP-hPST could classify the 30 compounds with 97% accuracy, 100% specificity and 93% sensitivity. Compared with metabolic biomarker-based hPSC assay by Stemina, the µP-hPST could successfully identify misclassified drugs Bosentan, Diphenylhydantoin and Lovastatin, and show a higher accuracy and sensitivity. This scalable µP-hPST may serve as either an independent assay or a complement assay for existing assays to reduce animal use, accelerate early discovery-phase drug screening and help general chemical screening of human teratogens.

## Introduction

The decreasing fertility rate and increasing age of pregnant women make healthy pregnancy a top priority for global health^1, 2^. Exposure to teratogenic compounds during pregnancy can disrupt normal embryo development and cause early embryo death, growth retardation, or severe birth defects. Currently most companies still rely on *in vivo* animal tests for predicting potential teratogens, which have been considered as the regulatory gold standard for decades^3^. However, due to large number of animals required and high inter-species variations in compound responses^4^, these costly and time-consuming animal tests are usually performed at a very late stage of compound development in at least two species and the general concordance to human responses is not very satisfactory^5^. Therefore, alternative *in vitro* human pluripotent stem cells (hPSCs)-based tests with reasonable accuracy should be developed and validated to establish regulatory standards^6^.

Currently available hPSC-based models, together with those animal cell-based models, mainly focus on recapitulating temporal disruptions of cell proliferation and/or differentiation by teratogenic compounds using various molecular biomarkers such as proteins^7, 8^, genes^9^, activity of specific signaling pathways^10^ or metabolites^6^. However, *in vivo* embryo development is a very complex process, which involves not only cell proliferation and differentiation, but also series of morphogenic movements spatially and temporally^11–13^. Disruption of cell proliferation and/or differentiation may only partially identify teratogenic effects of test compounds. Previously we have developed the first *in vitro* human development model mimicking spatiotemporally controlled mesoendoderm development using micropatterning, where both cell differentiation and cell migration were modeled and quantified^14^. A confluent monolayer of circular hPSC colonies were patterned onto the tissue culture surface, and exposed to mesoendoderm induction medium for 3 days. Due to higher mechanical stress at the colony periphery compared with interior, cells at the periphery would differentiate into mesoendoderm cells^15^ and migrate inwards to form a consistent mesoendoderm pattern on day 3^14^. When dosing with paradigm teratogens, any disruptions on normal cell differentiation and collective cell migration could be captured by quantifying the morphology changes of the formed mesoendoderm patterns. A proof-of-concept testing using four known teratogens and one non-teratogen showed its improvement of teratogen detection compared to the mouse embryonic stem cell test (mEST).

In order to further develop this *in vitro* micropatterned hPSC (μP-hPSC) model for industrial applications, here we optimized and validated the assay with a group of 30 compounds. We acquired the cytotoxicity profile of each compound on both human embryonic cell line H9 cells and adult human dermal fibroblasts (aHDFs), and tested the dose response of each compound in the µP-hPSC colonies under mesoendoderm development. After acquiring the fluorescent images of mesoendoderm marker Brachyury (T) of the µP-hPSC colonies, we identified the most sensitive morphological features to reflect the major compound-induced mesoendoderm pattern changes. The lowest disruption concentration (LDC) of each compound was identified based on the reduced features. We also developed a two-step classification rule for the teratogen identification to establish the *in vivo* relevance of the compound screening in our micropatterned human pluripotent stem cell test (µP-hPST). This refined and validated assay with high predictability for teratogen detection would find wide applications in not only drug development but also as a cost-saving assay for early detection of teratogens in industrial chemicals, household and consumer goods, food and nutraceuticals, cosmetics and environmental toxins. This human cell-based *in vitro* assay will resolve the inter-species variation problems of the existing assays and contribute greatly to the reduction of animal uses.

## Results

### Cytotoxicity profile of test compounds

**Figure 1:**
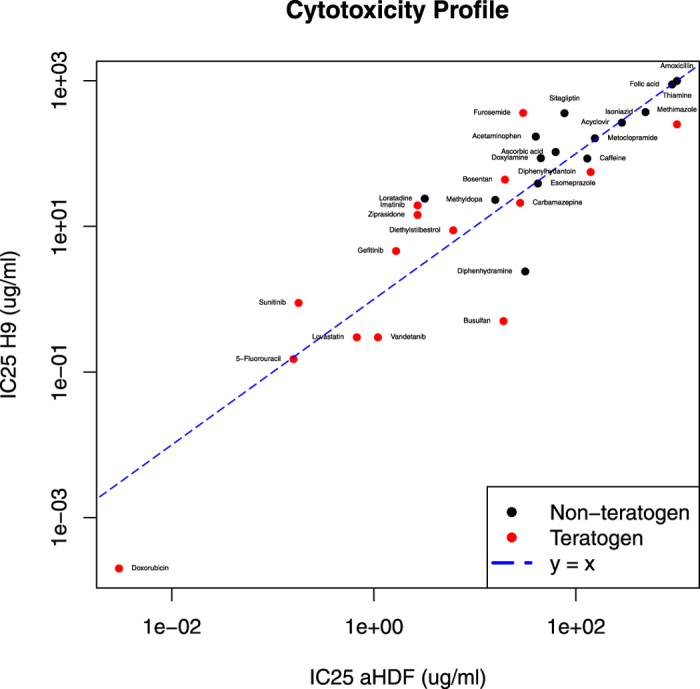
Cytotoxicity profile of test compounds Red: teratogens, Black: non-teratogens.

A set of 30 compounds of known teratogenicity was selected to establish and validate the µP-hPST (Table 1). The compounds include equal number of non-teratogens and teratogens, and differ in chemical property and therapeutic usage. Before tested in the µP-hPSC colonies, the cytotoxicity profile of each compound was acquired. Two cell lines, hPSC line H9 and adult human dermal fibroblast line aHDF, were tested to represent embryonic cytotoxicity and general cytotoxicity of the compound respectively (Supplementary Figs S1, S2). The IC_25_ values, which were the 25% inhibitory concentrations, were calculated as the highest non-cytotoxic concentrations of the compound (Table 2, Fig. 1). Generally, compounds which were toxic at very low concentrations were mostly teratogens, such as Doxirubicin, 5-Fluorouracil, Sunitinib and Lovastatin (IC_25_ values < 1 µg/ml); while compounds which showed minimum toxicity were mostly non-teratogens, such as Amoxicillin, Folic acid and Thiamine (IC_25_ values > 800 µg/ml) (Fig. 1). Similarity in cytotoxicity profile could be found in compounds belonging to the same class of chemical property or therapeutic use, especially in terms of general cytotoxicity. Antineoplastic drugs (5-Fluorouracil, Diethylstilbestrol, Doxorubicin and tyrosine kinase inhibitors (TKIs)) showed general cytotoxicity at relatively low concentrations (IC_25aHDF_ < 10 µg/ml) compared with other classes. Three of the four tested TKIs (Gefitinib, Imatinib and Sunitinib) exhibited higher general cytotoxicity than embryonic cytotoxicity, similar to antihypertensive compounds Methyldopa and Bosentan. Ethanolamine-based Histamine-1 antagonist Diphenhydramine and Doxylamine had similar general cytotoxicity (IC_25aHDF_ = 31.4 vs 45 µg/ml), but varied greatly in terms of embryonic cytotoxicity (IC_25H9_ = 2.4 vs 87 µg/ml). Loratadine, a piperidine Histamine H1 receptor antagonist, on the other hand, showed a much higher general cytotoxicity than Diphenhydramine and Doxylamine (IC_25aHDF_ = 3.2 µg/ml). While Vitamins Folic acid (Vitamin B9) and Thiamine (Vitamin B1) exhibited minimal toxicity to cells, Ascorbic acid (Vitamin C) showed toxicity at a much lower concentration and exhibited higher general cytotoxicity (IC_25aHDF_ = 63 µg/ml < IC_25H9_ = 105 µg/ml). No obvious correlation was observed between compounds’ teratogenicity potential and their higher embryonic cytotoxicity. There was similar number of compounds that had higher embryonic cytotoxicity than their general cytotoxicity in both non-teratogens and teratogens (5 VS 8, Fig. 1).

**Table 1:** Description of test compounds. The chemical properties and usages of drugs were found in PubChem Open Chemistry Database (https://pubchem.ncbi.nlm.nih.gov). NON: Non-teratogen, TER: Teratogen.

Drug name	Chemical property	Therapeutic use	FDA label	*In vivo* teratogenicity
Acetaminophen	Derivative of acetanilide	Analgesic and antipyretic	B	NON
Acyclovir	Nucleoside analog, DNA polymerase Inhibitor.	Antiviral, to treat herpes labialis and genital herpes	B	NON
Amoxicillin	Penicillin	Antibiotic	B	NON
Ascorbic acid	Vitamin C	To treat or prevent vitamin C deficiency	A	NON
Caffeine	Methylxanthine, adenosine receptor antagonist, phosphodiesterase inhibitor	Central nervous system stimulant, anti-inflammatory	C	NON
Diphenhydramine	Ethanolamine-based Histamine-1 antihistamine	Antiemetic, antitussive, anti-allergic, hypnotic and sedative	B	NON
Doxylamine	Ethanolamine-based Histamine-1 antihistamine	To treat allergy symptoms and insomnia; also used as an antitussive and antiemetic	A	NON
Esomeprazole	Proton pump inhibitor	Anti-ulcer, gastric acid secretion inhibitor and astrointestinal agent	B	NON
Folic acid	Vitamin B9	To treat or prevent folate deficiencies and megaloblastic anemia	A	NON
Isoniazid	Synthetic derivative of nicotinic acid	Antitubercular	C	NON
Loratadine	Piperidine Histamine H1 receptor antagonist	Antipruritic and anti-allergic, to treat allergic rhinitis and urticaria	B	NON
Metoclopramide	Dopamine D2 antagonist	Gastroprokinetic and antiemetic	B	NON
Methyldopa	Adrenergic alpha2-Agonist	Antihypertensive	B	NON
Sitagliptin	Dipeptidyl peptidase 4 inhibitor	Hypoglycemic, to treat type 2 diabetes	B	NON
Thiamine	Vitamin B1	To treat or prevent vitamin B1 deficiency	A	NON
5-Fluorouracil	Nucleoside metabolic inhibitor	Antineoplastic	D	TER
Bosentan	Endothelin receptor antagonist	Antihypertensive, to treat pulmonary artery hypertension	X	TER
Busulfan	Alkylating agent	Anti-Leukemia	D	TER
Carbamazepine	Tricyclic compound chemically related to tricyclic antidepressants (TCA)	Anticonvulsant and analgesic	D	TER
Diethylstilbestrol	Non-steroidal oestrogen hormone	Antineoplastic, to treat breast and prostate cancer	X	TER
Diphenylhydantoin	Hydantoin derivative	Anticonvulsant	D	TER
Doxorubicin	Anthracycline topoisomerase inhibitor	Antineoplastic	D	TER
Furosemide	Sulfamoylanthranilic acid derivative	Diuretic; to treat hypertension and edema	C	TER
Gefitinib	Tyrosine kinase inhibitor, EGFR inhibitor	Antineoplastic, to treat lung cancer	D	TER
Imatinib	Tyrosine kinase inhibitor, inhibiting Bcr-Abl fusion protein tyrosine kinase, PDGFR and SCF/c-kit	Anti-Leukemia and antineoplastic, to treat dermatofibrosarcoma protuberans and malignant gastrointestinal stromal tumors	D	TER
Lovastatin	HMG-CoA reductase inhibitor	Anticholesteremic	X	TER
Methimazole	Thyroid hormone synthesis inhibitor	Antithyroid, to treat hyperthyroidism	D	TER
Sunitinib	Tyrosine kinase inhibitor, inhibiting VEGFR2, PDGFRb, c-kit, and FLT3	Antineoplastic, to treat gastrointestinal stromal tumor, pancreatic neuroendocrine tumor, and advanced renal cell carcinoma	D	TER
Vandetanib	Tyrosine kinase inhibitor, inhibiting VEGFR2 and EGFR	Antineoplastic, to treat symptomatic or progressive medullary thyroid cancer	D	TER
Ziprasidone	Benzothiazolylpiperazine derivative; antagonist at the dopamine D2, serotonin 5-HT2A, 5-HT1A, 5-HT1D receptors;	Antipsychotic, to treat Bipolar I disorder and Schizophrenia	C	TER

**Table 2:** Compound screening results in µP-hPST. The C_max_ values of each compound were mainly acquired from U.S. Food and Drug Administration website, European Medicines Agency website or published journals unless otherwise noted. The full list could be found in Supplementary Table S1. NON: Non-teratogen, TER: Teratogen, NA: not applicable.

Drug name	IC_25aHDF_ (µg/ml)	IC_25H9_ (µg/ml)	C_max_ (µg/ml)	LDC (µg/ml)	Step1: Is LDC <= IC_25 aHDF_ & IC_25H9?_	Step 2: Is LDC <= 10 C_max?_	Teratogenicity in µP-hPST	Teratogenicity in other *in vitro* assays	*In vivo* teratogenicity
Acetaminophen	40	171	24	160	N	-	NON	NON^6^	NON
Acyclovir	285	267	5.65	60	Y	N	NON	NON^6, 7^	NON
Amoxicillin	>1000	>1000	14.4	150	Y	N	NON	NON^6^	NON
Ascorbic acid	63	105	156	80	N	—	NON	NON^6^	NON
Caffeine	129	85.4	10	130	N	—	NON	NON^6, 7^	NON
Diphenhydramine	31.4	2.4	0.06	2.4	Y	N	NON	NON^6^	NON
Doxylamine	45	87	0.12	45	Y	N	NON	NON^6^	NON
Esomeprazole	42.1	38.9	1.62	45	N	—	NON	NON^7^	NON
Folic acid	894	888	0.27	900	N	—	NON	NON^6^	NON
Isoniazid	488	370	3.09	500	N	—	NON	NON^6^	NON
Loratadine	3.2	24	0.04	4	N	—	NON	NON^6^	NON
Metoclopramide	154	162	0.04	120	Y	N	NON	NON^6^	NON
Methyldopa	15.9	23.2	7.5	25	N	—	NON	NON^7^	NON
Sitagliptin	77.1	359	0.39	>360	N	—	NON	NON^6^	NON
Thiamine	>1000	>1000	0.11	1000	Y	N	NON	NON^6^	NON
5-Fluorouracil	0.16	0.15	48.4	0.025	Y	Y	TER	TER^6, 7^	TER
Bosentan	19.9	43.8	8.17	16	Y	Y	TER	NON^6^	TER
Busulfan	19.2	0.5	1.22	0.2	Y	Y	TER	TER^6^	TER
Carbamazepine	28	21	11	10	Y	Y	TER	TER^6^	TER
Diethylstilbestrol	6.1	8.8	0.0056	5	Y	N	NON	TER^7^	TER
Diphenylhydantoin	140	56	20	50	Y	Y	TER	NON^6^	TER
Doxorubicin	0.003	0.0002	0.37	0.00015	Y	Y	TER	TER^7^	TER
Furosemide	30	362	400	30	Y	Y	TER	NON^28^	TER
Gefitinib	1.66	4.6	0.93	0.9	Y	Y	TER	TER^7^	TER
Imatinib	2.7	19.5	1.56	0.75	Y	Y	TER	TER^7^	TER
Lovastatin	0.68	0.30	0.01	0.01	Y	Y	TER	NON^6^	TER
Methimazole	>1000	252	0.3	1.5	Y	Y	TER	NA	TER
Sunitinib	0.18	0.89	0.03	0.15	Y	Y	TER	TER^7^	TER
Vandetanib	1.1	0.3	0.4	0.3	Y	Y	TER	TER^7^	TER
Ziprasidone	2.7	14.4	0.2	1.25	Y	Y	TER	TER^7^	TER

The cytotoxicity of compounds may interfere with their teratogenic effects and disrupt the mesoendoderm pattern formation in µP-hPSC colonies. Therefore, the IC_25aHDF_ and IC_25H9_ values of each compound served as the cytotoxicity thresholds for teratogen testing and classification in our µP-hPST.

### Dose-dependent mesoendoderm pattern disruptions by test compounds

The key test of the µP-hPST is to examine the morphological disruption effects of teratogenic compounds on µP-hPSC colonies during mesoendoderm formation. In normal mesoendoderm induction medium without compound exposure, cells at the periphery of the µP-hPSC colonies would always differentiate into T^+^ mesoendoderm cells and migrate inwards to form a consistent annular mesoendoderm pattern after 3 days of culture^14^. Teratogen would affect the cell differentiation and/or migration process and change the morphology of the mesoendoderm patterns in a dose-dependent manner. To explore the teratogenic potential of test compounds, we selected 6 concentrations of each compound and tested them on the µP-hPSC colonies. The concentrations were selected to cover compound’s cytotoxicity thresholds (IC_25aHDF_ and IC_25H9_) as well as its highest therapeutic concentration in human plasma (C_max_) (Table 2, Supplementary Table. S1). The µP-hPSC colonies were incubated in mesoendoderm induction medium together with the test compounds for 3 days. The medium was half changed every day. On day 3, all the colonies were fixed and immunostained for mesoendoderm marker T, the distribution of which indicated the morphology of the mesoendoderm patterns (Supplementary Fig. S3).

**Figure 2:**
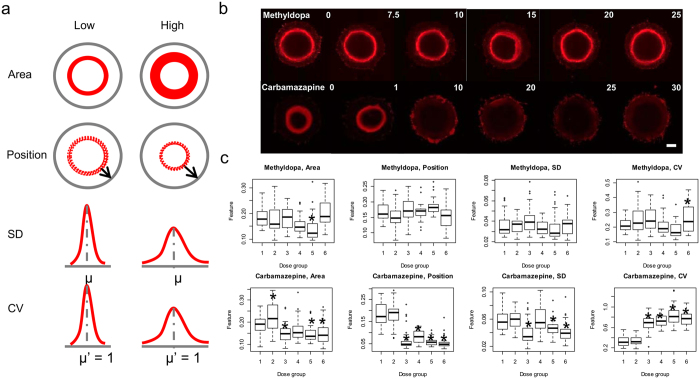
Morphological features showing the dose-dependent response of mesoendoderm pattern disruption. (**a**) The four features extracted from T fluorescent images. SD: Standard deviation; CV: Coefficient of variation. µ, µ′: Mean. (**b**) Representative T fluorescent images after drug treatment. Methyldopa: non-teratogen; Carbamazepine: teratogen. Scale bar, 200 µm. (**c**) Boxplots of the morphological features after Methyldopa and Carbamazepine treatment. Dose groups (1–6): 0, 7.5, 10, 15, 20, 25 μg/ml for Methyldopa, 0, 1, 10, 20, 25, 30 μg/ml for Carbamazepine. *p < 0.01. The LDC for Methyldopa and Carbamazepine are 25 µg/ml and 10 µg/ml respectively.

From the T fluorescent images, we could see that most non-teratogens showed no obvious disruption effects compared with teratogens even at the highest dose except Acyclovir, Ascorbic acid, Diphenhydramine, Metoclopramide and Thiamine (Fig. 2b, Supplementary Fig. S3a,b). Most teratogens, on the contrary, were observed with a clear dose-dependent response within the test range (Fig. 2b, Supplementary Fig. S3c,d). Different disruption patterns could be identified from the images. The compounds could slow down or prevent mesoendoderm cell migration, for instance, in the case of Thiamine and Diphenylhydantoin; or promote cell migration, as in the case of Diethylstibestrol and Lovastatin. They could also decrease both cell differentiation and migration, as in the case of Carbamazepine; or promote both cell differentiation and migration, as in 5-Fluorouracil; or even promote mesoendoderm differentiation while decrease collective cell migration, as in Methimazole and Sunitinib. Beside teratogenicity, cytotoxicity effects could also affect mesoendoderm patterns. For example, under Busulfan treatment at concentrations higher than 0.5 μg/ml, which is its IC_25H9_ value, cell number in the µP-hPSC colonies decreased. Together with the compound’s migration acceleration effects, much smaller and concentrated mesoendoderm patterns were observed (Supplementary Fig. S3c). At concentration of 10 μg/ml, only a thin monolayer of cells remained within the colony and the mesoendoderm pattern was totally lost.

### Morphological feature selection to capture compounds’ disruption effects

Since various morphological disruption patterns were acquired after compound treatment, a quantitative, multi-variate image characterization method was needed to measure the disruption effects and identify the lowest disruption concentration (LDC) of each compound. Initially we extracted 19 morphological features from each T fluorescent image to characterize the spatial distribution of the mesoendoderm cells. They were the typical statistical data descriptors such as sum (area), mean (position), variance, kurtosis, etc. Four features were related to skewness and entropy that did not correlate with compound treatment^14^. Among the remaining 15 features, we clustered them into 7 morphological groups to quantify various compound disruptions. Plotting these compound disruption effects into box plots, we found that the high order statistical descriptors such as kurtosis and energy are less sensitive than the low order ones such as area, position, standard deviation (SD) and coefficient of variation (CV), in correlating with the compound-induced morphological changes at low concentrations. Therefore, only these four best morphological features were selected to quantitatively characterize the mesoendoderm patterns among groups (Fig. 2a).

To identify the LDC of a test compound, we plotted the values of these four features from all dose groups and compared with the untreated controls respectively using unpaired two sample t-tests (Fig. 2c, Supplementary Fig. S4). A significant disruption was defined as *p* < 0.01. The lowest concentration which showed a significant morphological disruption without a breaking point at higher concentrations in at least one of the four features would be defined as the LDC (Fig. 2c, Supplementary Fig. S4).

### Generation of a two-step teratogen classification method

**Figure 3:**
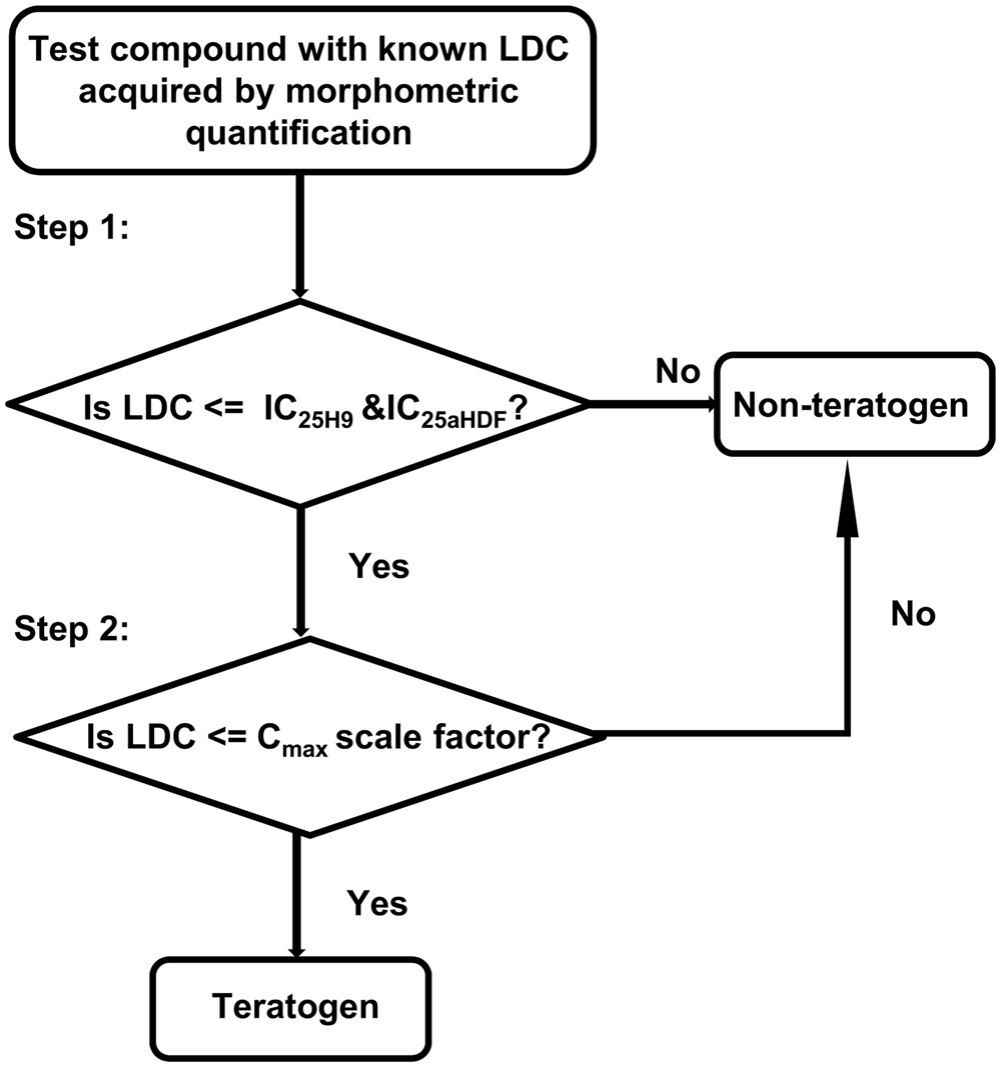
Two-step decision rule for teratogen classification.

After the LDC values of all test compounds were identified in µP-hPST (Table 2), compound classification was performed. Since the cytotoxicity of compounds may also disrupt the mesoendoderm pattern formation, we first compared the LDC values with the IC_25_ values of aHDF and H9 cells. The LDC of teratogens should be no higher than their IC_25aHDF_ and IC_25H9_ values. Otherwise, the test compound could be classified as a non-teratogen. By using this rule, 9 of the 30 compounds would be classified as non-teratogens and the remaining 21 compounds would be classified as teratogens (Table 2). All of the 9 non-teratogens were consistent with their *in vivo* classifications; however, 6 of the identified teratogens were misclassified. When comparing their LDC values with their respective C_max_ values, we found that the LDC values were much higher, indicating that the physiological relevance should also be taken into account. To achieve this, a second step of the classification was implemented to examine the *in vivo* relevance (Fig. 3). A threshold using a scale factor of C_max_ was applied. If the LDC of a test compound were higher than the scale factor of C_max_, which means this teratogenic effect was unlikely to happen clinically, we would classify the compound as a non-teratogen; otherwise, it be a teratogen (Fig. 3).

**Figure 4:**
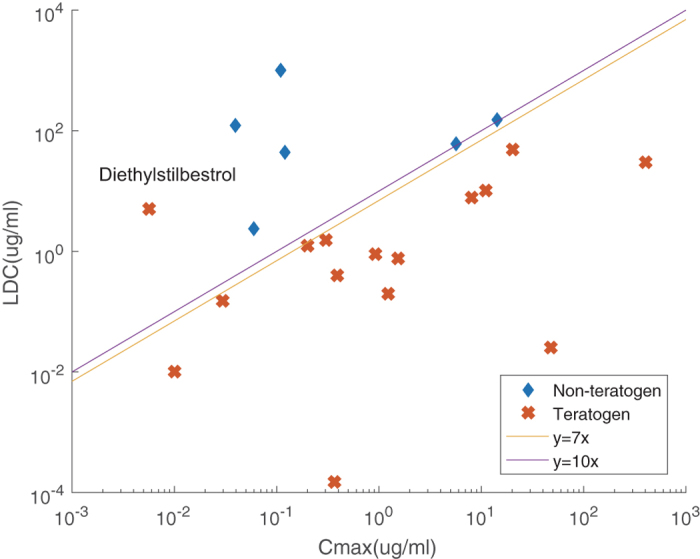
Linear comparison results for the identification of best C_max_ scale factor. Only Diethylstilbestrol was misclassified as a non-teratogen when Step 2 of the classification rule was LDC <= 7–10 fold of C_max_ for teratogens.

To investigate the value of scale factor for C_max_, we had all 21 unclassified compounds tested using leave-one-out rule after Step 1^16, 17^. The LDC and C_max_ values of the compounds, together with their known teratogenicity class served as inputs to the model to find the optimal scale factor (Supplementary Table S2). Results showed that a scale factor of 7–10 could achieve the best classification accuracy across all test runs (AUC >= 96%) (Supplementary Fig. S4), with only Diethylstilbestrol misclassified as false positive (Fig. 4, Table 2). To verify such optimal scale factor, we also had LDC and C_max_ used as features and thus created a binary classification model using multinomial logistic regression (MLR) and support vector machine (SVM). The binary classification results (AUC = 91.7% for both MLR and SVM) were comparable to the scale factor based classification with only one compound being misclassified (Supplementary Fig. S6) and thus proved that second step of proposed classification rule could recapitulate the inherent difference of the tested compounds.

### Assay performance

By extracting four morphological features to find LDC and a two-step classification method to classify the compounds, we could improve our µP-hPST assay to achieve 97% accuracy, 100% specificity and 93% sensitivity in screening 30 paradigm compounds (Table 3). We compared our assay performance with the Targeted Biomarker Assay^6^, which is one of the state-of-the-art hPSC-based teratogen screening models. The assay measures the ratio of two metabolites which are Ornithine and Cystine in hPSC maintenance culture to identify compound’s disruption concentration, and compares it with *in vivo* C_max_ to classify the compound’s teratogenicity^6^. The µP-hPST captures the disruption of mesoendoderm patterns by affecting spatiotemporally controlled cell differentiation and migration, and exhibits better performance in terms of accuracy, sensitivity and specificity. It could correctly classify Bosentan, Diphenylhydantoin and Lovastatin, which were all misclassified in the Targeted Biomarker Assay (Table 3). It can also correctly identify Furosemide as teratogen which was misclassified in mEST (Table 2).

**Table 3:** Summary of the uP-hPST assay performance.

Assay	No. of compounds	Sensitivity	Specificity	Accuracy
µP-hPST	30	93%	100%	97%
Targeted Biomarker Assay	36	79%	100%	89%

## Discussion

Precise control of cell differentiation and migration patterns is critical during all stages of the embryo development^18–21^. Teratogens could disrupt embryo development through interfering cell differentiation and morphogenetic movements, resulting in various birth defects. Currently one of the most accurate and commonly used clinical examinations for teratogenicity is direct morphological observations using B-mode ultrasonography. For *in vitro* teratogen screenings, some studies have examined the morphological changes due to teratogenic disruptions in both cell differentiation and migration using animal embryos or cells, such as the rat whole embryo culture (rWEC) assay^22^, the zebrafish embryo culture (ZEC) assay^23, 24^, and a newly developed assay using mouse embryoid body (mEB) by Marikawa’s group^25, 26^. However, these assays are under performing (~70–80% accuracy for the rWEC and ZEC assays)^27^. We have established the first *in vitro* hPSC-based and fully quantitative morphometric assay which captured both cell differentiation and cell migration processes, and successfully eliminated inter-species variations^14^. Refined and validated using 30 compounds, our µP-hPST exhibited the highest accuracy compared with other existing *in vitro* teratogen screening models using animal embryos and cells^8, 10, 22, 24, 27, 28^, or hPSCs^6, 7^.

The µP-hPST recapitulated the mesoendoderm formation process which occurs during gastrulation. One key reason is that the spatial and temporal control of primitive streak formation and mesoendoderm generation is critical in initiating the correct human body plan, which is followed by the three germ layer generation and organogenesis^29, 30^. By quantifying the morphological disruptions of the cell differentiation and collective cell migration patterns that are the two critical processes in every stage of the embryonic development, we could detect teratogens affecting not just mesoendoderm but also the other stages of embryo development. For example, the µP-hPST can detect the teratogenic effects of Diphenylhydantoin and Carbamazepine. Diphenylhydantoin is known to induce orofacial, limb, cardiovascular and neurobehavioral anomalies^6, 31^, and Carbamazepine could cause neural tube defects, cardiovascular and urinary tract anomalies, and cleft palate in newborns^32^.

Dose-dependent response is a key feature of compounds’ teratogenic effects. The identification of human teratogens always correlates with their clinical therapeutic dose. Therefore, it’s crucial to compare the *in vitro* teratogenic concentration range with their *in vivo* therapeutic dose when screening teratogens *in vitro*. In this study, without comparing the *in vitro* LDC with the *in vivo* C_max_ of the test compounds, the assay would yield high false positive rate (6/21 = 28.6%). The second step of the classification approach was established based on an intuitive linear relationship assumption between LDC and C_max_, and verified using MLR and SVM binary classification models with comparable classification accuracy. After applying a second classification step which excluded compounds whose LDC was higher than 7–10 fold of C_max_, only one false negative result was observed. This two-step classification rule was also applicable to the five paradigm compounds tested previously^14^, all the compounds (one known non-teratogen and four teratogens) could still be correctly classified (Supplementary Table S3). For predictability of teratogen identification among unknown compounds, the two-step classification method has its unique advantages compared with the Targeted Biomarker Assay, which uses C_max_ as a reference for classification^6^. For compounds with unknown C_max_, the µP-hPST can quickly identify candidates which are more likely non-teratogens by acquiring all negatives in Step 1 of the classification rule, and provide useful information regarding the potential *in vivo* teratogenic concentration range for those with uncertain teratogenicity.

The µP-hPST achieved 97% accuracy using the two-step classification method in the current teratogen screen of 30 compounds, among which the identified teratogens could affect different stages of embryonic development with different mechanisms of teratogenicity. However, considering the complexity of the teratogenic mechanisms, more teratogen screens using additional compounds are required to further test the predictability of our current assay. These compounds may include chemicals of different chemical classes and usages, or compounds which have differential teratogenic effects but share similar chemical structures.

Although *in vivo* animal assays have been used for teratogen screening for decades and are required by regulatory bodies, they cannot fully reproduce human responses due to high inter-species variations (~40%)^5^. False negatives generated by these *in vivo* animal models due to species variability have caused irreversible social tragedies such as the case of Thalidomide^33^. An increase from testing one animal species to two could lower the chance of false negatives to ~12.5% (by keeping the 40% species variability consistent), but would significantly increase the false positive rate^5^. Our µP-hPST is an *in vitro* hPSC-based assay which eliminates the inter-species variability, and has much better prediction accuracy (97% accuracy, 100% specificity and 93% sensitivity) compared with both *in vivo* animal models and other existing *in vitro* assays. However, it still cannot detect all teratogenic compounds possibly due to the inherent limitations shared by all *in vitro* assays (1 in 15 teratogens was classified as false negative). A combination of the µP-hPST with a second *in vivo* or *in vitro* assay may serve as an option for human teratogen detection. For compounds with existing animal testing data, the use of µP-hPST could eliminate potential false negatives by providing human-specific testing results. As for new compounds screening, the µP-hPST could be first applied to save costs and time, and comparing the negatives with further *in vivo* animal test. The µP-hPST could best serve as an early-phase teratogen screening assay for pharmaceutical companies and research institutions, or a late phase verification of animal testing results.

## Conclusion

The µP-hPST is the first *in vitro* hPSC-based morphometric assay for human teratogen screening, which could capture compound’s teratogenic effects in both cell differentiation and collective cell migration. It eliminated inter-species variations, and achieved 100% specificity, 93% sensitivity and 97% accuracy in 30 compound screen. The µP-hPST could serve as a good teratogen-screening assay independently or complementing other assays to reduce animal use, accelerate early discovery-phase drug screening, or general chemical screening.

## Materials and Methods

### Compounds

All compounds used in this study were purchased from Selleck Chemicals or Sigma-Aldrich: 5-Fluorouracil (F6627,Sigma-Aldrich), Acetaminophen (A7085, Sigma-Aldrich), Acyclovir (S1807, Selleck Chemicals), Amoxicillin (S3015, Selleck Chemicals), Ascorbic acid (A5960, Sigma-Aldrich), Bosentan (S4220, Selleck Chemicals), Busulfan (B2635, Sigma-Aldrich), Caffeine (27600, Sigma-Aldrich), Carbamazepine (C8981, Sigma-Aldrich), Diethylstilbestrol (D4528, Sigma-Aldrich), Diphenhydramine (S1866, Selleck Chemicals), Diphenylhydantoin (D4007, Sigma-Aldrich), Doxorubicin (S1208, Selleck Chemicals), Doxylamine (D3775, Sigma-Aldrich), Esomeprazole (S1743, Selleck Chemicals), Folic acid (F7876, Sigma-Aldrich), Furosemide (F4381, Sigma-Aldrich), Gefitinib (S1025, Selleck Chemicals), Imatinib (S1026, Selleck Chemicals), Isoniazid (S1937, Selleck Chemicals), Loratadine (S1358, Selleck Chemicals), Lovastatin (S2061, Selleck Chemicals), Methimazole (S1609, Selleck Chemicals), Methyldopa (S1642, Selleck Chemicals), Metoclopramide (M0763, Sigma-Aldrich), Sitagliptin (S4002, Selleck Chemicals), Sunitinib (S1042, Selleck Chemicals), Vandetanib (S1046, Selleck Chemicals), Thiamine (T4625, Sigma-Aldrich), Ziprasidone (S1444, Selleck Chemicals).

All compounds were dissolved in DMSO (D2650, Sigma-Aldrich) except the following compounds, which were dissolved in culture medium: Acyclovir, Caffeine, Methimazole, Isoniazid, Folic acid and Thiamine.

### Cell maintenance and differentiation

Human embryonic cell line H9 (WiCell Research Institute, Inc) and adult human dermal fibroblast (aHDF, Lonza) were cultured as previously described^14^. Briefly, H9 cells were cultured in tissue culture plates coated with Matrigel^TM^ (354277, BD Biosciences) in mTeSR^TM^1 medium (05850, StemCell^TM^ Technologies). Dispase (07923, StemCell^TM^ Technologies) treatment and mechanical scraping were applied during normal subculture. The aHDF cells was cultured in DMEM medium (10569-010, Gibco), supplemented with 10% FBS (SV30160.03, Thermo Scientific Hyclone) and 1% Pen-Strep (09367-34, Nacalai Tesque). Cells were subcultured every 5–6 days using 0.25% Trypsion-EDTA (25200-114, Gibco) to detach the cells.

To induce mesoendodermal differentiation, H9 cells were cultured in serum-free STEMdiff^TM^ APEL^TM^ medium (05210, StemCell^TM^ Technologies) supplemented with 100 ng/ml Activin A (338-AC-025, R&D Systems), 25 ng/ml BMP4 (314-BP-010, R&D Systems) and 10 ng/ml FGF2 (233-FB-025, R&D Systems). For compound testing in the μP-hPST, the final concentration of DMSO in the medium was lower than 0.5%.

### Cytotoxicity assay

The adverse effect of test compounds on cell proliferation and viability was detected using CellTiter 96® AQueous One Solution Cell Proliferation Assay (MTS, G3580, Promega). 10,000 H9 cells or 500 aHDF cells were seeded in 96-well tissue culture plates. For H9 cells, Matrigel^TM^ was coated before seeding. For each compound, 8 concentrations from serial 5-fold dilutions were tested besides the vehicle controls. Cells were cultured for 3 days with medium half change every day before cell viability was measured. Each drug was tested in three independent runs and its IC_25_ values (inhibitory concentrations which can cause 25% of reduction in cell number) were acquired by logistic regression using OriginPro 9.

### Generation of μP-hPSC colonies

The polydimethylsiloxane (PDMS) stencil was fabricated as previously described^14^. Circular patterns with 1 mm in diameter were generated on each stencil, which is in square shape and fit for 60-mm dishes. To generate μP-hPSC colonies, autoclaved PDMS stencils were sealed onto tissue culture dishes using 70% ethanol, and coated with 300 μl Matrigel^TM^ diluted in DMEM/F12 (11330032, GIBCO) for 5 hr at 37 °C. For cell seeding, single cells were acquired using Accutase (SCR005, Merck Millipore), and seeded at 5190 cells/mm^2^ in cell maintenance medium containing 10 μM Y27632 (688000, Calbiochem, Merck Millipore). After 45 min, stencils were peeled off and the surface was treated with 0.5% Pluronic F-127 (P2443, Sigma-Aldrich) in DMEM/F12 for passivation. Cells were incubated for at least 3 hr in Y27632-containing medium before switched to normal mTeSR^TM^1 medium. Differentiation started the next day.

### Immunofluorescence staining and microscopy

On day 3 of differentiation, cells were fixed using 4% paraformaldehyde for immunofluorescence staining of mesoendoderm marker Brachyury (T). Cells were permeabilized with 0.5% Triton X-100 in 1X PBS and incubated in 2% BSA in 0.1% PBS Triton X-100 for 3 hr at room temperature (RT). Cells were incubated overnight with goat anti-Brachyury (AF2085, R&D Systems) primary antibody (1:100) at 4 °C and then incubated in Alexa Fluor^®^ 546 donkey anti-goat IgG (A11056, Life Technologies) secondary antibody (1:1000) for 1 hr at RT after washing. Fluorescence imaging was done using Olympus IX81 epifluorescence microscope (Olympus, Japan).

### Morphological feature extraction

Image analysis was performed using Matlab Image Processing Toolbox and Statistical Toolbox (Mathworks). The µP-hPSC colonies were identified using intensity differences from background. Segmentation using Otsu’s method was performed to identify T^+^ regions of the colonies. Four morphologic features including Area, Position, Standard deviation (SD) and Coefficient of variation (CV) of the T^+^ region were calculated to represent the morphological distribution of the mesoendoderm patterns.

### Classification model

All the classification analysis was done using Matlab Statistical Toolbox (Mathworks). To identify the best scale factor of C_max_ for drug classification, linear comparison was performed using LDC and C_max_ values. The leave-one-out cross validation rule was applied. For each run, a factor ranging from 1 to 20 was tested with an interval of 1. Best parameters were then chosen based on the highest area under the receiver operating characteristic curve (AUC) values acquired in respective Receiver Operating Curve (ROC) evaluation. Multinomial logistic regression (MLR) and support vector machine (SVM) using leave-one-out cross validation approach were further utilized to verify the classification accuracy of linear comparison using LDC and C_max_ as input features. The performances were evaluated using their respective AUC values.

### Statistical analysis

The statistical analysis was done in R (Version 3.1.2). Unpaired two-sample t-test was performed to evaluate statistical differences between compound-treated groups and control group. Differences were deemed as significant if *p* < 0.01. The first dose showing significant disruptions without breakpoint in the following higher concentrations will be determined as the LDC of test compound.

## Electronic supplementary material

Supplementary Information
