# Supplementary material for: In Vitro Micropatterned Human Pluripotent Stem Cell Test (µP-hPST) for Morphometric-Based Teratogen Screening
Source: Sci Rep. 2017 Aug 17;7:8491. doi: 10.1038/s41598-017-09178-1 (PMC5561212; doi:10.1038/s41598-017-09178-1)
Supplement: Supplementary file 1 — Supplementary Information [file 41598_2017_9178_MOESM1_ESM.pdf]

Supplementary Information

In Vitro Micropatterned Human Pluripotent Stem Cell Test (μP-hPST) for Morphometric-Based Teratogen Screening

Jiangwa Xing[1*], Yue Cao[1,2], Yang Yu[1,3,4], Huan Li[1], Ziwei Song[1,4] and Hanry Yu[1-5*]

[1] Institute of Bioengineering and Nanotechnology, A*STAR, The Nanos, #04-01, 31 Biopolis Way, Singapore 138669, Singapore
[2] Mechanobiology Institute, National University of Singapore, T-Lab, #05-01, 5A Engineering Drive 1, Singapore 117411, Singapore
[3] Singapore-MIT Alliance for Research and Technology, 1 CREATE Way, #10-01 CREATE Tower, Singapore 138602, Singapore
[4] Department of Physiology, Yong Loo Lin School of Medicine, MD9-04-11, 2 Medical Drive, Singapore 117597, Singapore
[5] Gastroenterology Department, Southern Medical University, Guangzhou 510515, China

* Corresponding authors:
Jiangwa Xing (Email: jwxing@ibn.a-star.edu.sg; Tel: +65 6824 7180 Fax: +65 6478 9080)
Hanry Yu (Email: hanry_yu@nuhs.edu.sg; Tel: +65 6824 7000 Fax: +65 6478 9080)

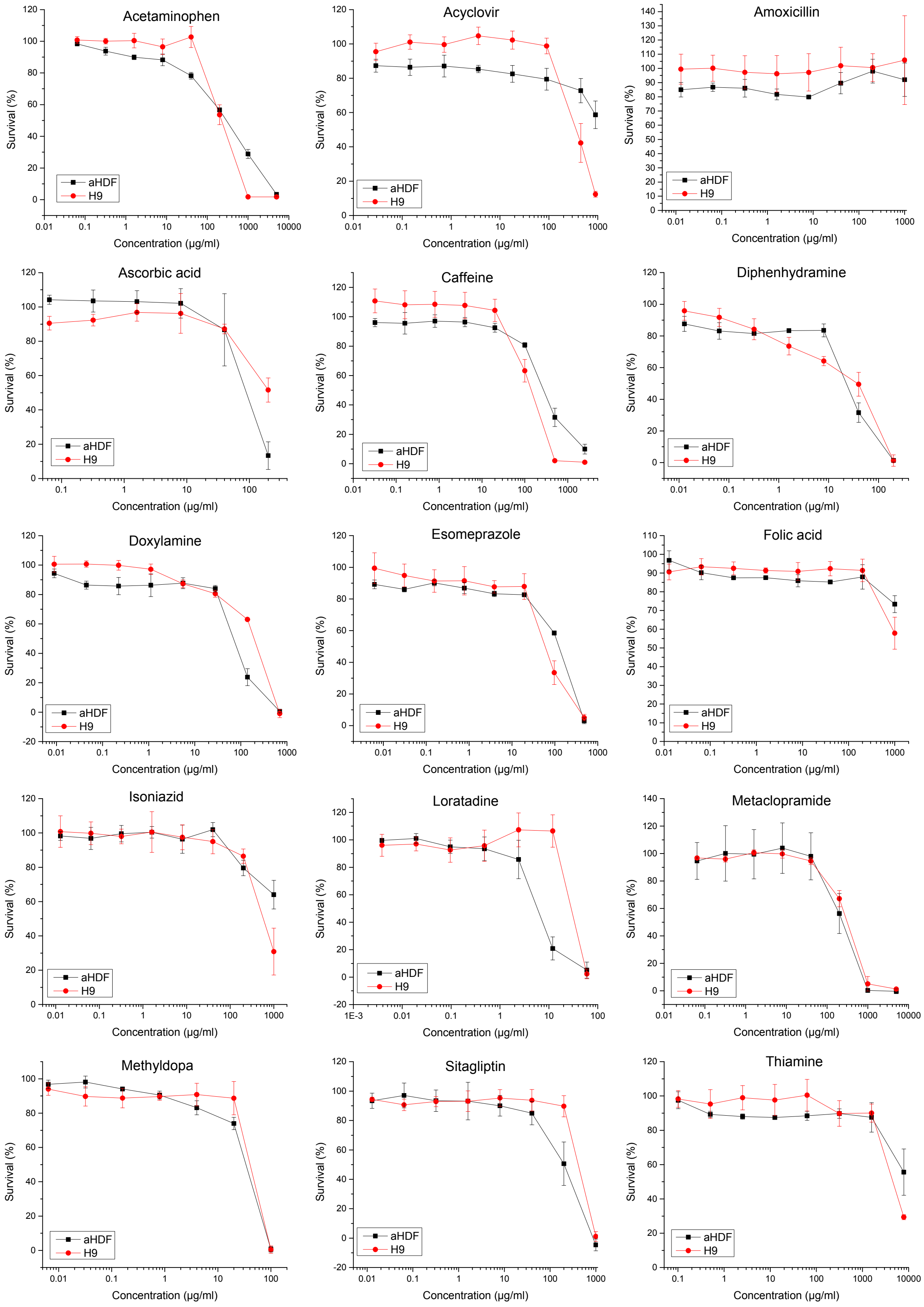

Figure S1 Cytotoxicity profile of non-teratogens

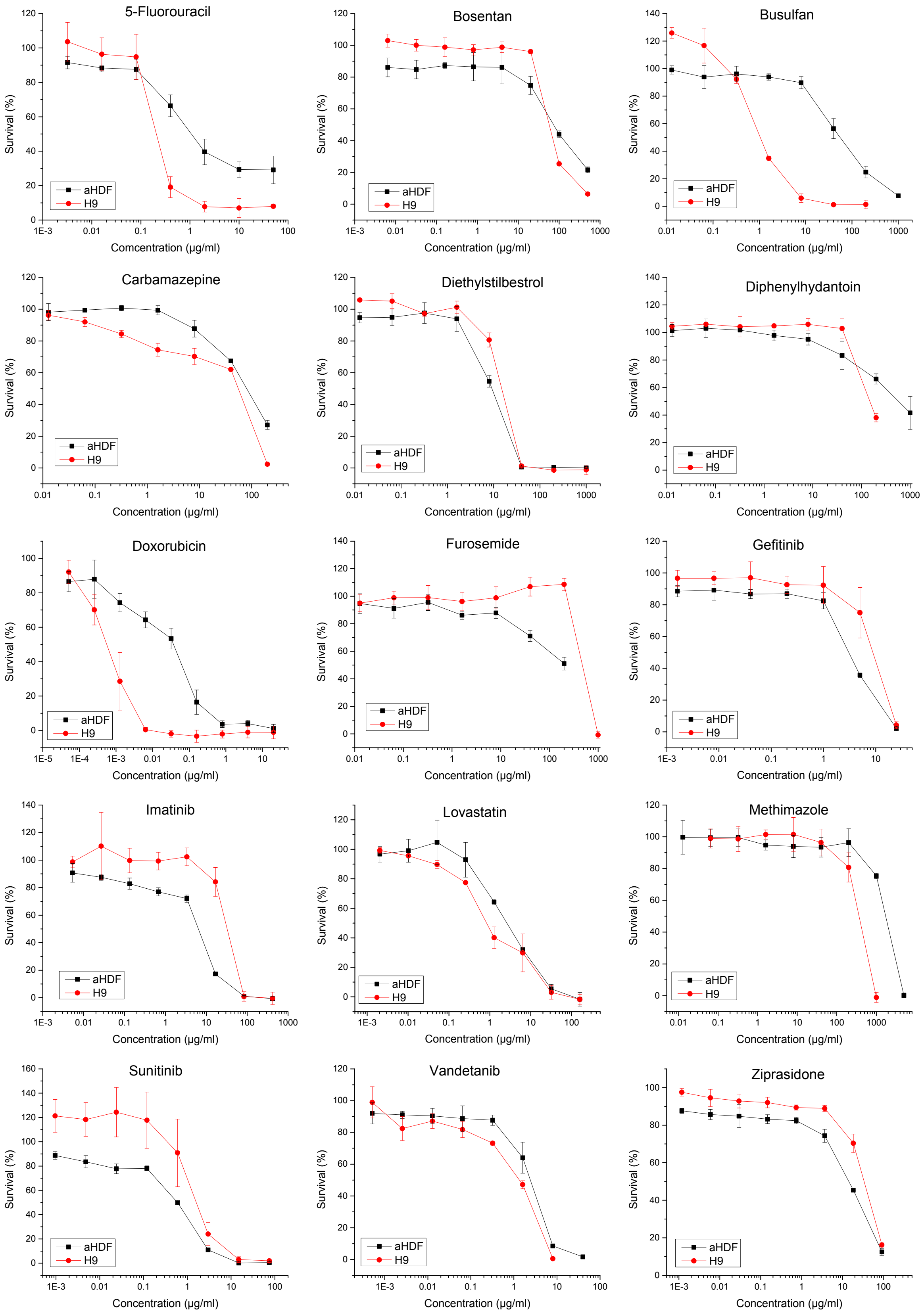

Figure S2 Cytotoxicity profile of teratogens

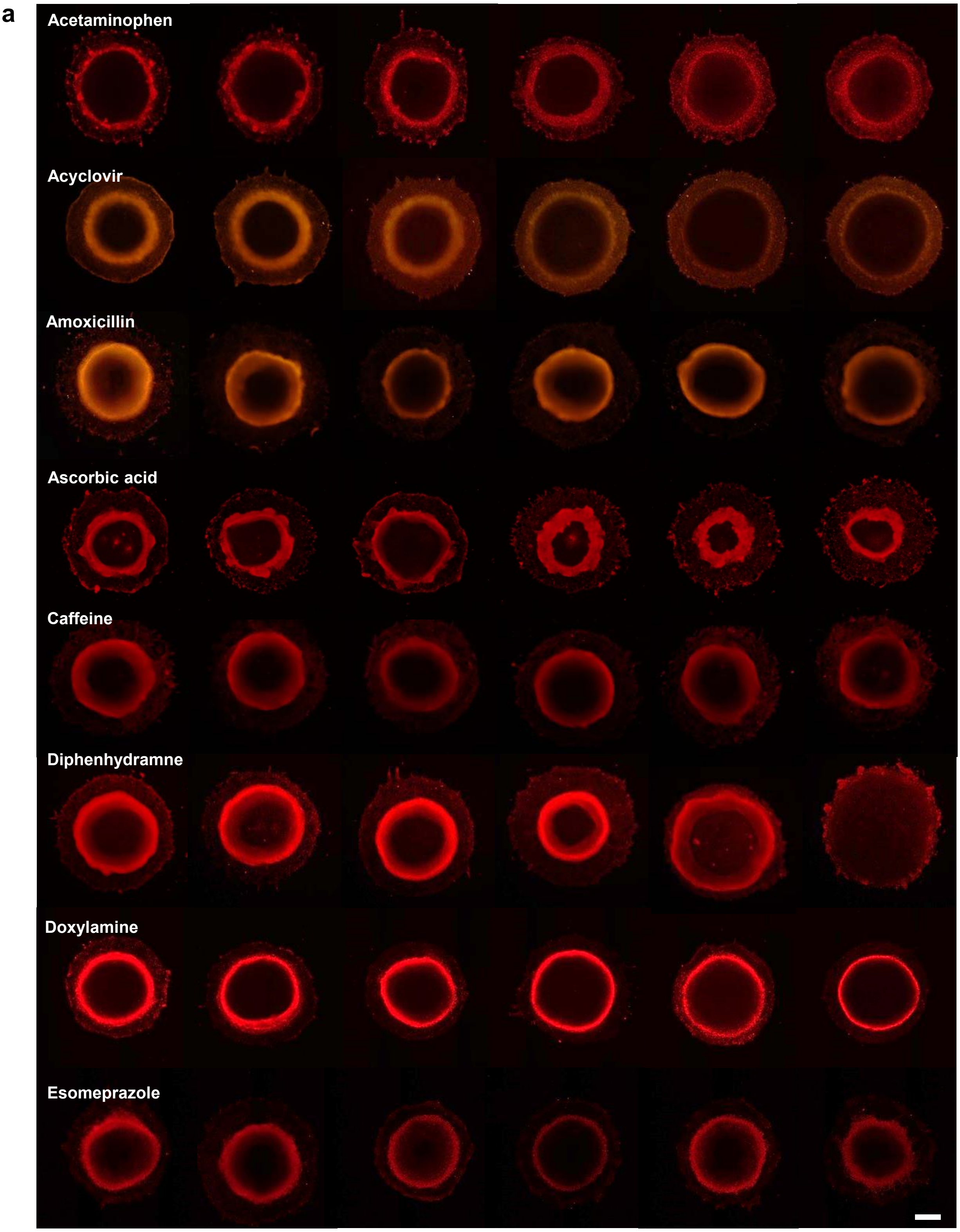

Figure S3 T Fluorescent images of μP-hPSC colonies under compound treatment. (a) and (b) refer to known non-teratogens, (c) and (d) refer to known teratogens. For each compound, the dose increases from left to right. (a) The specific doses are: 0, 20, 40, 80, 160, 180 μg/ml for Acetaminophen; 0, 6, 30, 60, 240, 300 μg/ml for Acyclovir; 0, 15, 60, 120, 150, 240 μg/ml for Amoxicillin; 0, 40, 60, 80, 100, 160 μg/ml for Ascorbic acid; 0, 10, 50, 85, 100, 130 μg/ml for Caffeine; 0, 0.06, 0.6, 2.4, 6, 30 μg/ml for Diphenhydramine; 0, 0.1, 10, 45, 80, 100 μg/ml for Doxylamine; 0, 1.6, 16, 35, 40, 45 μg/ml for Esomeprazole. Scale bar, 200 μm.

b

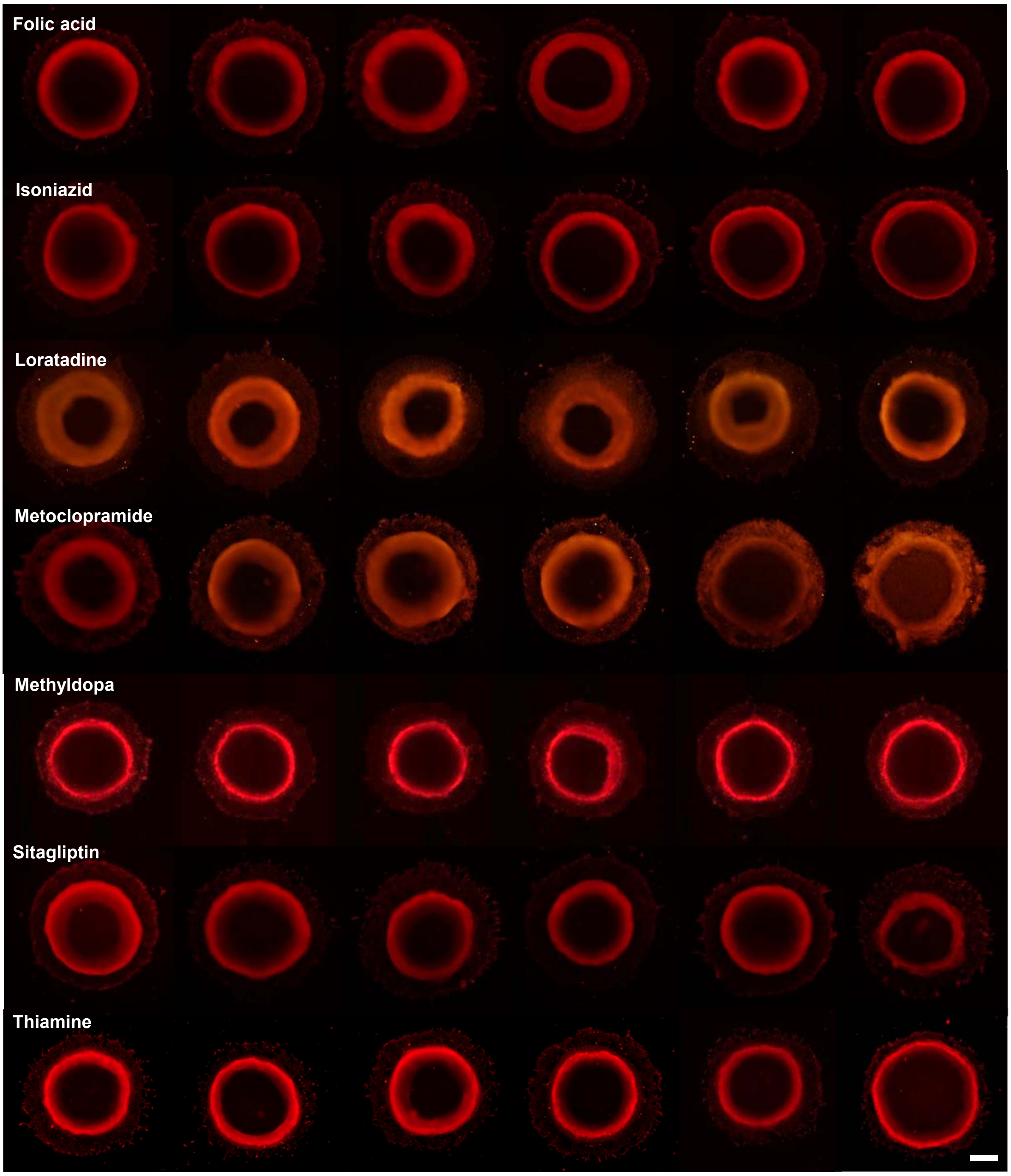

Figure S3 T Fluorescent images of μP-hPSC colonies under compound treatment (Continued). (b) The specific doses are: 0, 0.3, 3, 30, 300, 900 μg/ml for Folic acid; 0, 3, 30, 300, 400, 500 μg/ml for Isoniazid, 0, 0.04, 0.2, 0.4, 2, 4 μg/ml for Loratadine; 0, 0.04, 0.4, 4, 120, 150 μg/ml for Metoclopramide; 0, 7.5, 10, 15, 20, 25 μg/ml for Methyldopa; 0, 0.4, 4, 40, 80, 360 μg/ml for Sitagliptin; 0, 0.1, 10, 100, 500, 1000 μg/ml for Thiamine. Scale bar, 200 μm.

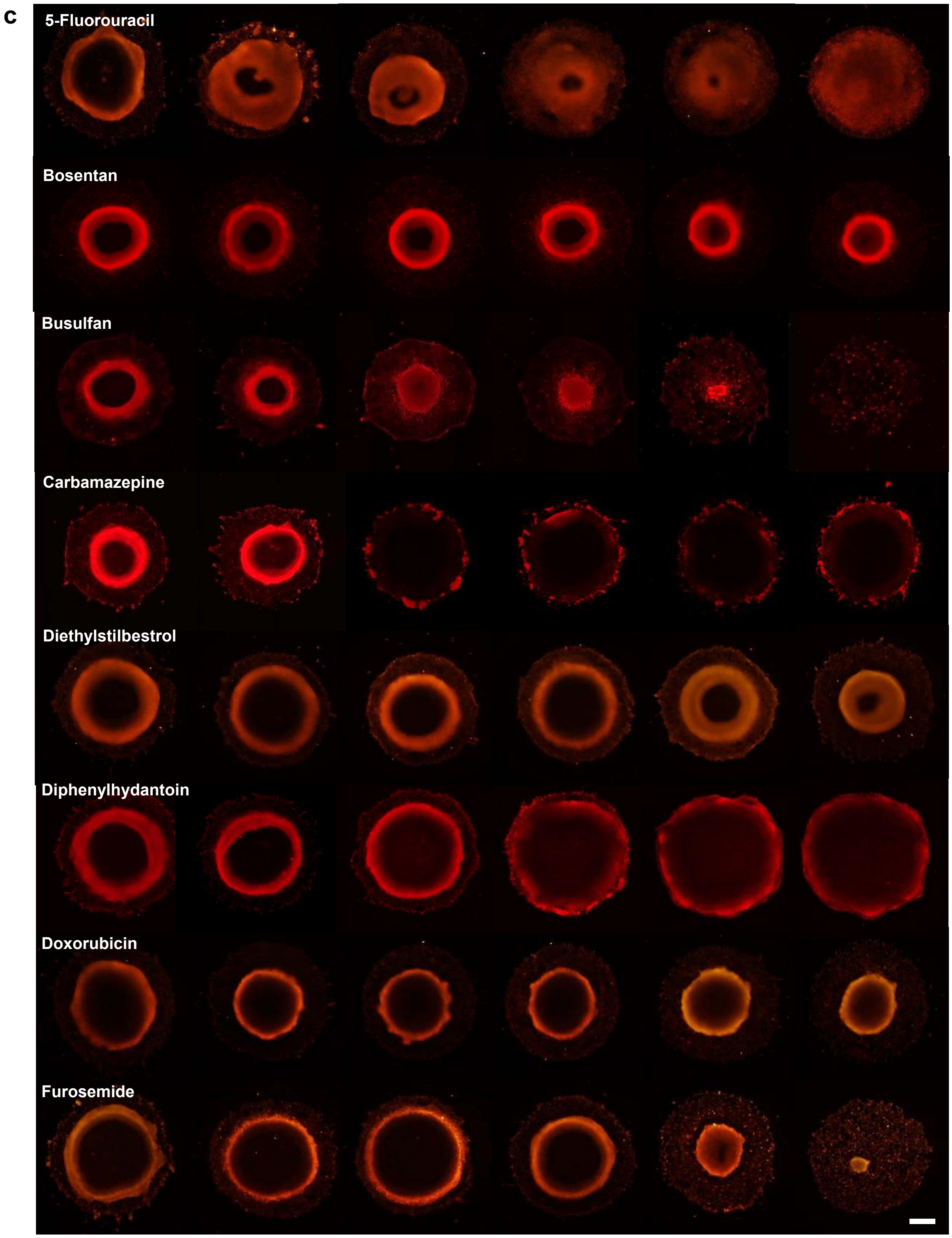

Figure S3 T Fluorescent images of μP-hPSC colonies under compound treatment (Continued). (c) The specific doses are: 0, 0.025, 0.05, 0.1, 0.15, 0.2 μg/ml for 5-Fluorouracil; 0, 8, 16, 20, 40, 45 μg/ml for Bosentan; 0, 0.2, 0.5, 1, 2, 10 μg/ml for Busulfan; 0, 1, 10, 20, 25, 30 μg/ml for Carbamazepine; 0, 0.005, 0.01, 0.05, 0.5, 5 μg/ml for Diethylstilbestrol, 0, 10, 20, 50, 100, 150 μg/ml for Diphenylhydantoin; 0, 0.0001, 0.00015, 0.0002, 0.002, 0.003 μg/ml for Doxorubicin; 0, 6, 30, 150, 300, 400 μg/ml for Furosemide; 0, 0.9, 1.5, 3, 4.5, 5 μg/ml for Gefitinib; 0, 0.75, 1.5, 2.3, 2.7, 10 μg/ml for Imatinib. Scale bar, 200 μm.

d

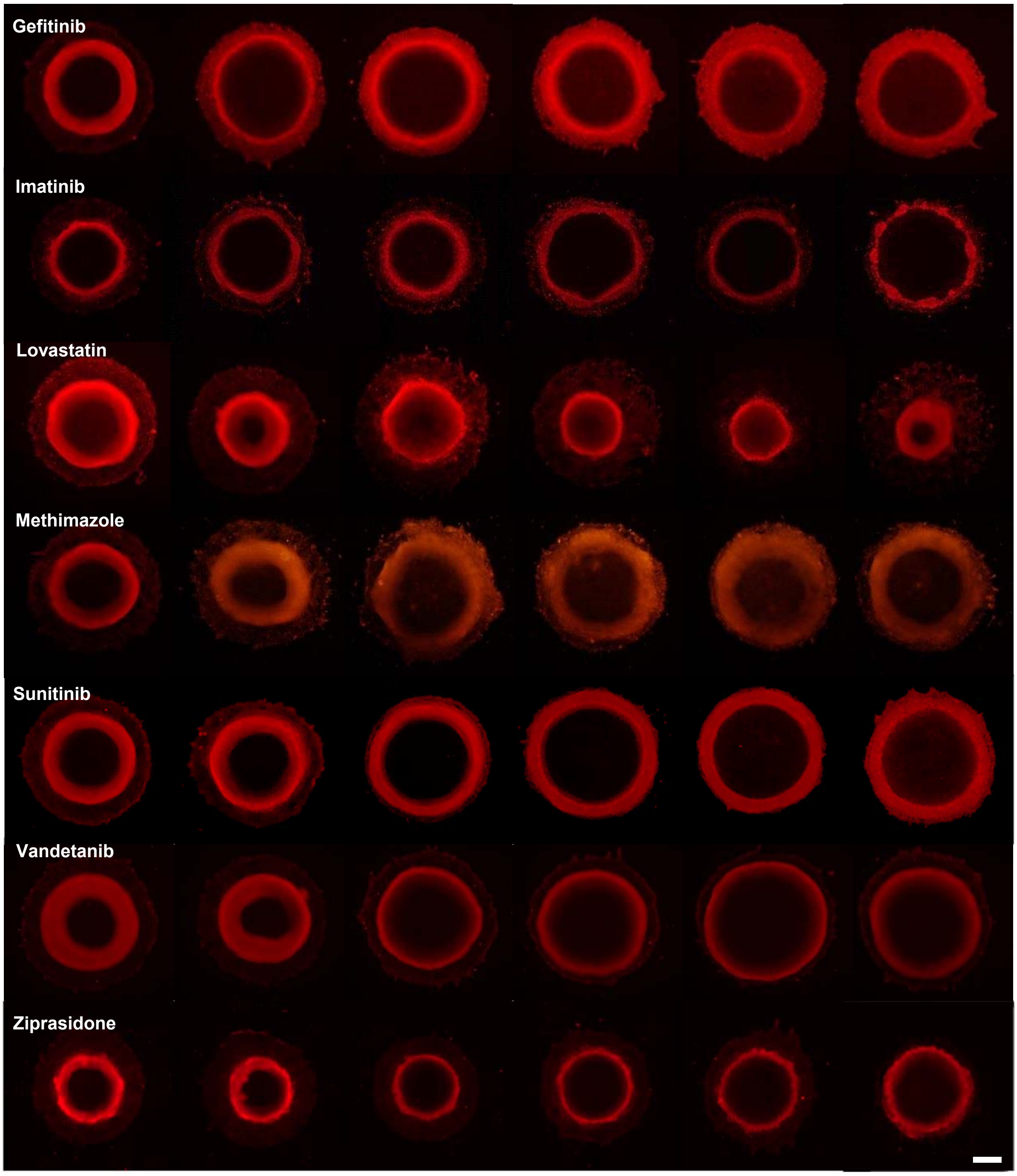

Figure S3 T Fluorescent images of µP-hPSC colonies under compound treatment (Continued). (d) The specific doses are: 0, 0.9, 1.5, 3, 4.5, 5 µg/ml for Gefitinib; 0, 0.75, 1.5, 2.3, 2.7, 10 µg/ml for Imatinib, 0, 0.01, 0.1, 0.2, 0.4, 0.8 µg/ml for Lovastatin; 0, 0.3, 1.5, 3, 30,150 µg/ml for Methimazole; 0, 0.03, 0.15,0.18, 0.54, 0.9 µg/ml for Sunitinib; 0, 0.2, 0.3, 0.4, 0.5, 1 µg/ml for Vandetanib; 0, 0.2, 1.25, 2.5, 5, 15 µg/ml for Ziprasidone. Scale bar, 200 µm.

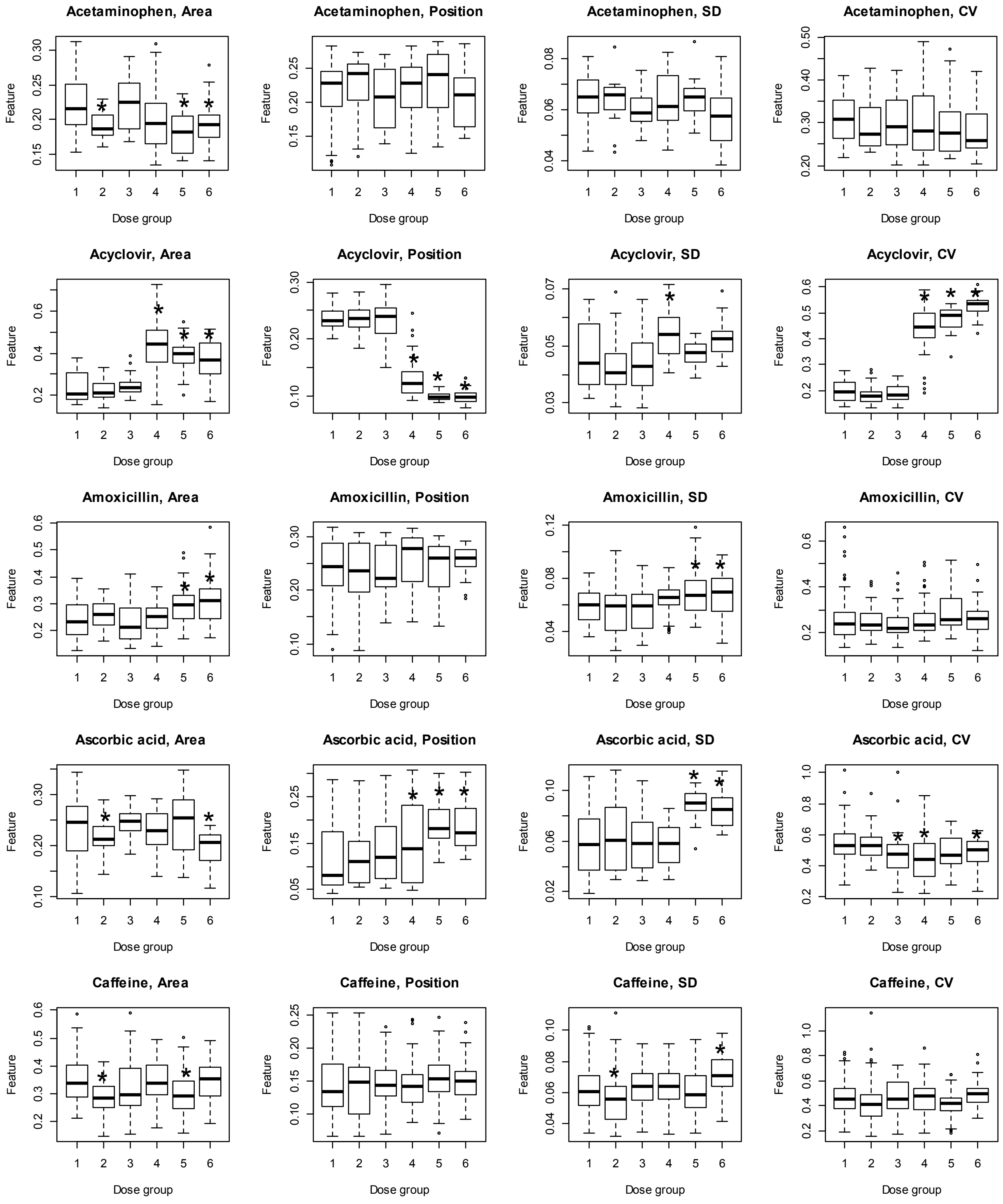

Figure S4 Boxplot of morphologic features for LDC detection. Dose groups (1-6): 0, 20, 40, 80, 160, 180 μg/ml for Acetaminophen; 0, 6, 30, 60, 240, 300 μg/ml for Acyclovir; 0, 15, 60, 120, 150, 240 μg/ml for Amoxicillin; 0, 40, 60, 80, 100, 160 μg/ml for Ascorbic acid; 0, 10, 50, 85, 100, 130 μg/ml for Caffeine. *: $p<0.01$.

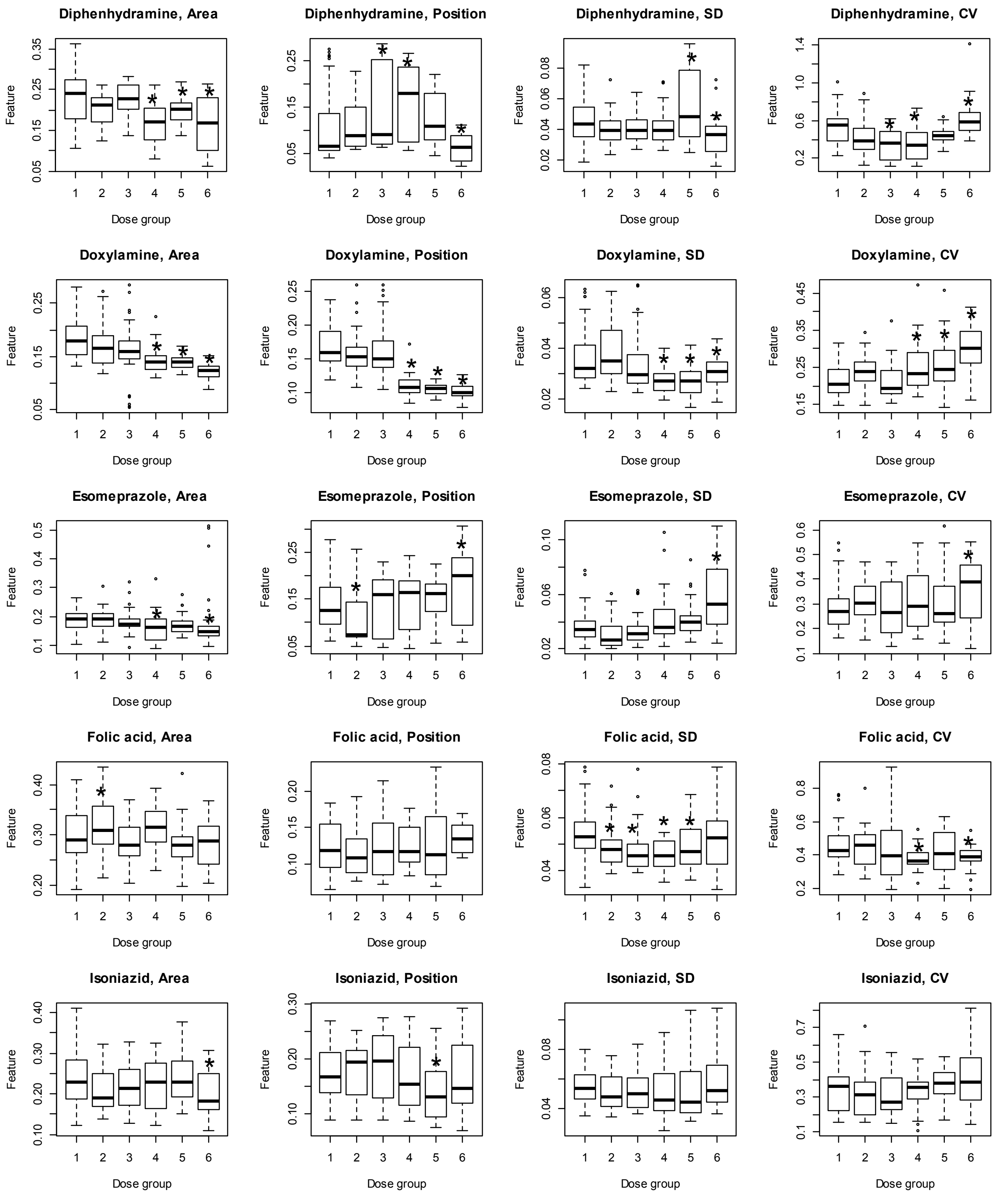

Figure S4 Boxplot of morphologic features for LDC detection (Continued)
Dose groups (1-6): 0, 0.06, 0.6, 2.4, 6, 30 μg/ml for Diphenhydramine; 0, 0.1, 10, 45, 80, 100 μg/ml for Doxylamine; 0, 1.6, 16, 35, 40, 45 μg/ml for Esomeprazole; 0, 0.3, 3, 30, 300, 900 μg/ml for Folic acid; 0, 3, 30, 300, 400, 500 μg/ml for Isoniazid. *: $p<0.01$.

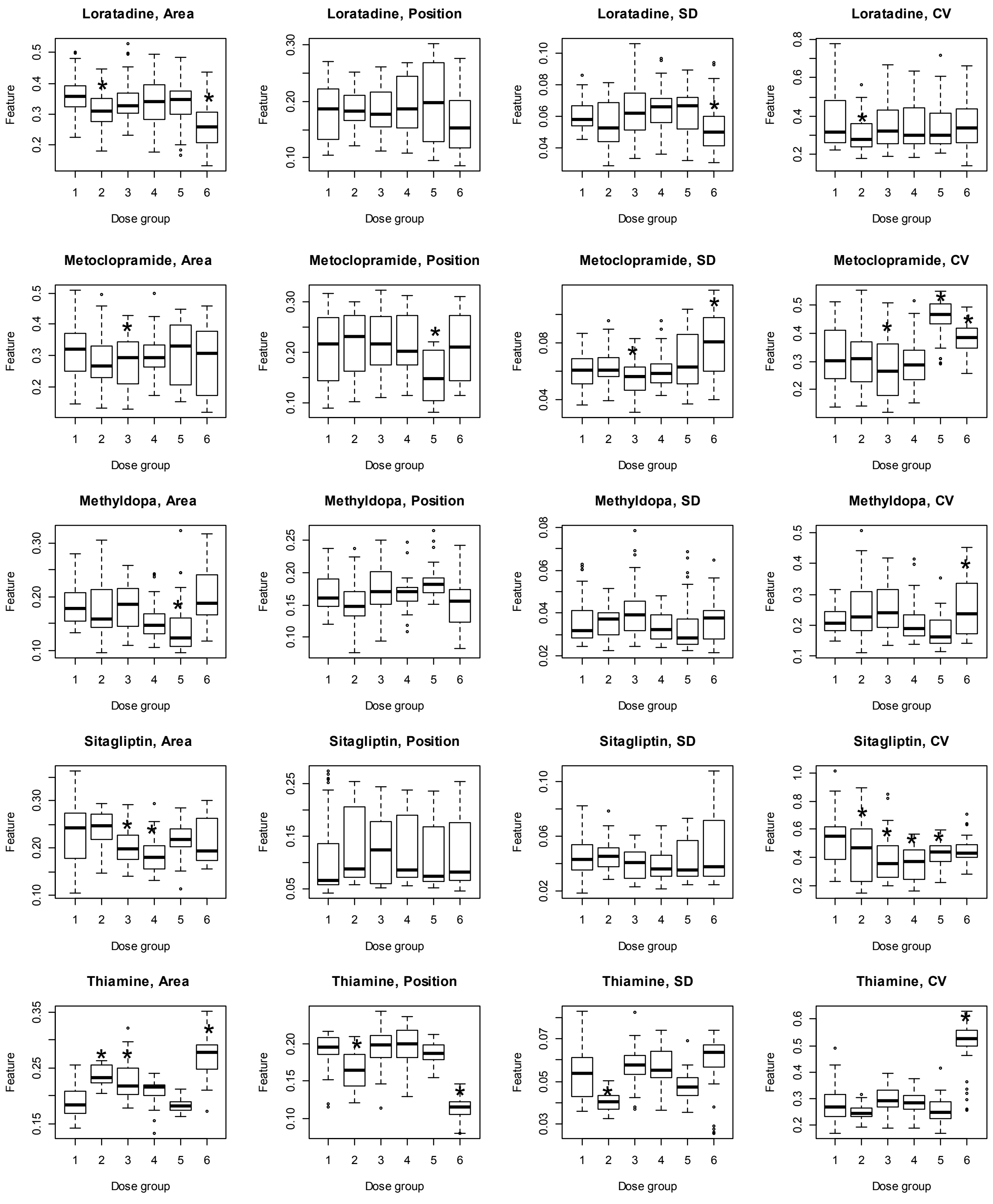

Figure S4 Boxplot of morphologic features for LDC detection (Continued)
Dose groups (1-6): 0, 0.04, 0.2, 0.4, 2, 4 μg/ml for Loratadine; 0, 0.04, 0.4, 4, 120, 150 μg/ml for Metoclopramide; 0, 7.5, 10, 15, 20, 25 μg/ml for Methyldopa; 0, 0.4, 4, 40, 80, 360 μg/ml for Sitagliptin; 0, 0.1, 10, 100, 500, 1000 μg/ml for Thiamine.
*: $p<0.01$.

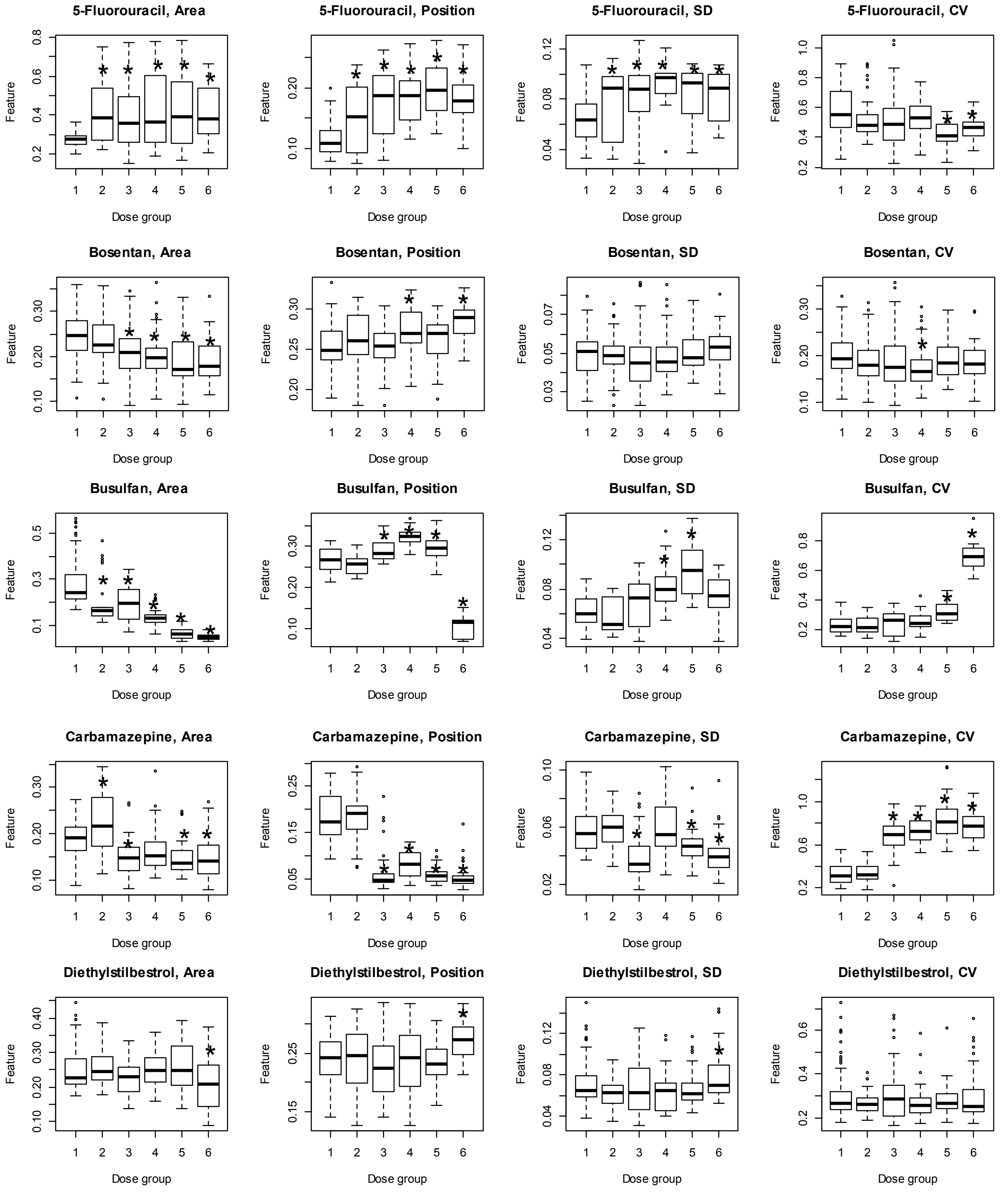

Figure S4 Boxplot of morphologic features for LDC detection (Continued)
Dose groups (1-6): 0, 0.025, 0.05, 0.1, 0.15, 0.2 μg/ml for 5-Fluorouracil; 0, 8, 16, 20, 40, 45 μg/ml for Bosentan; 0, 0.2, 0.5, 1, 2, 10 μg/ml for Busulfan; 0, 1, 10, 20, 25, 30 μg/ml for Carbamazepine; 0, 0.005, 0.01, 0.05, 0.5, 5 μg/ml for Diethylstilbestrol. *: $p<0.01$.

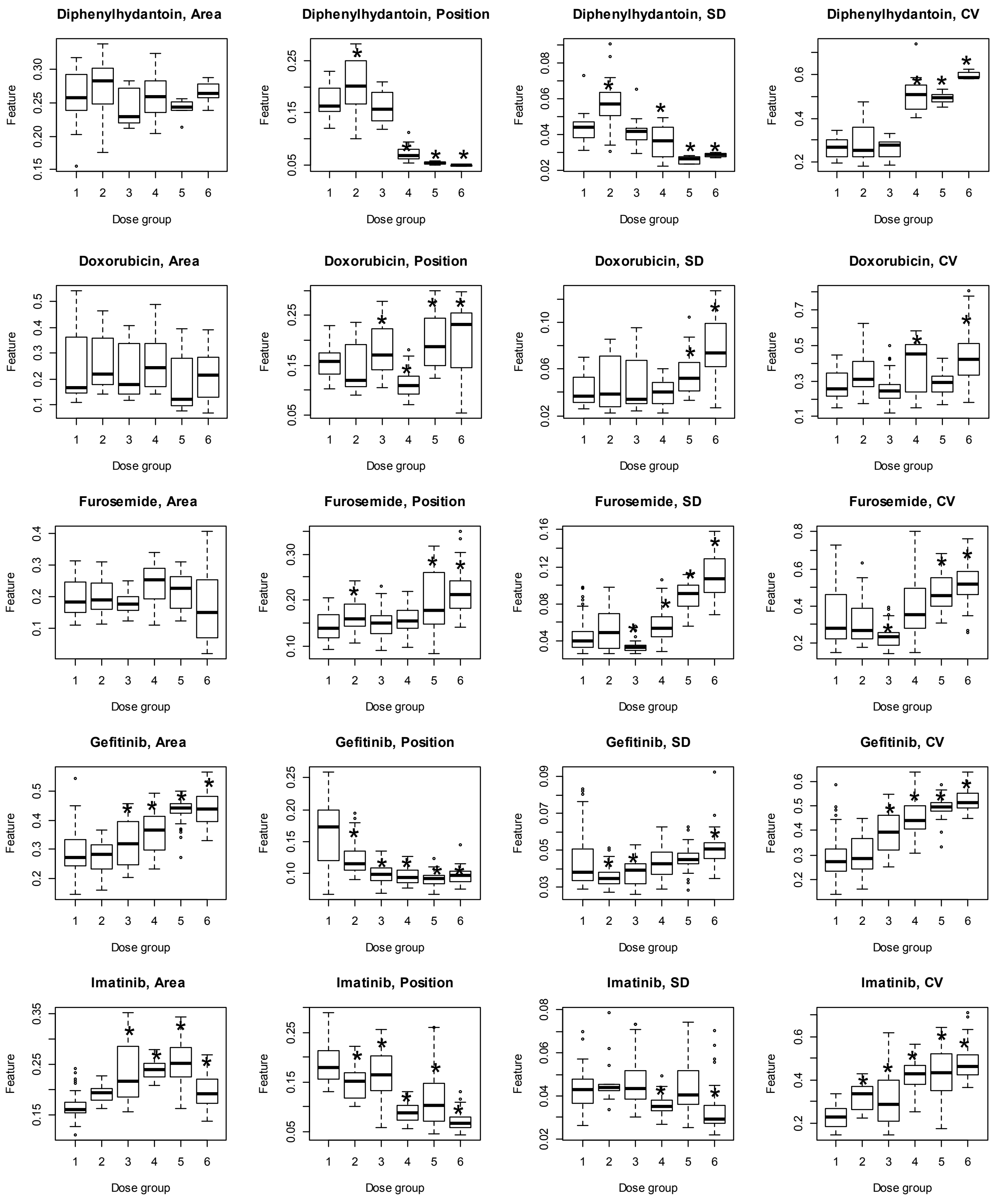

Figure S4 Boxplot of morphologic features for LDC detection (Continued)
Dose groups (1-6): 0, 10, 20, 50, 100, 150 μg/ml for Diphenylhydantoin; 0, 0.0001, 0.00015, 0.0002, 0.002, 0.003 μg/ml for Doxorubicin; 0, 6, 30, 150, 300, 400 μg/ml for Furosemide; 0, 0.9, 1.5, 3, 4.5, 5 μg/ml for Gefitinib; 0, 0.75, 1.5, 2.3, 2.7, 10 μg/ml for Imatinib. *: p<0.01.

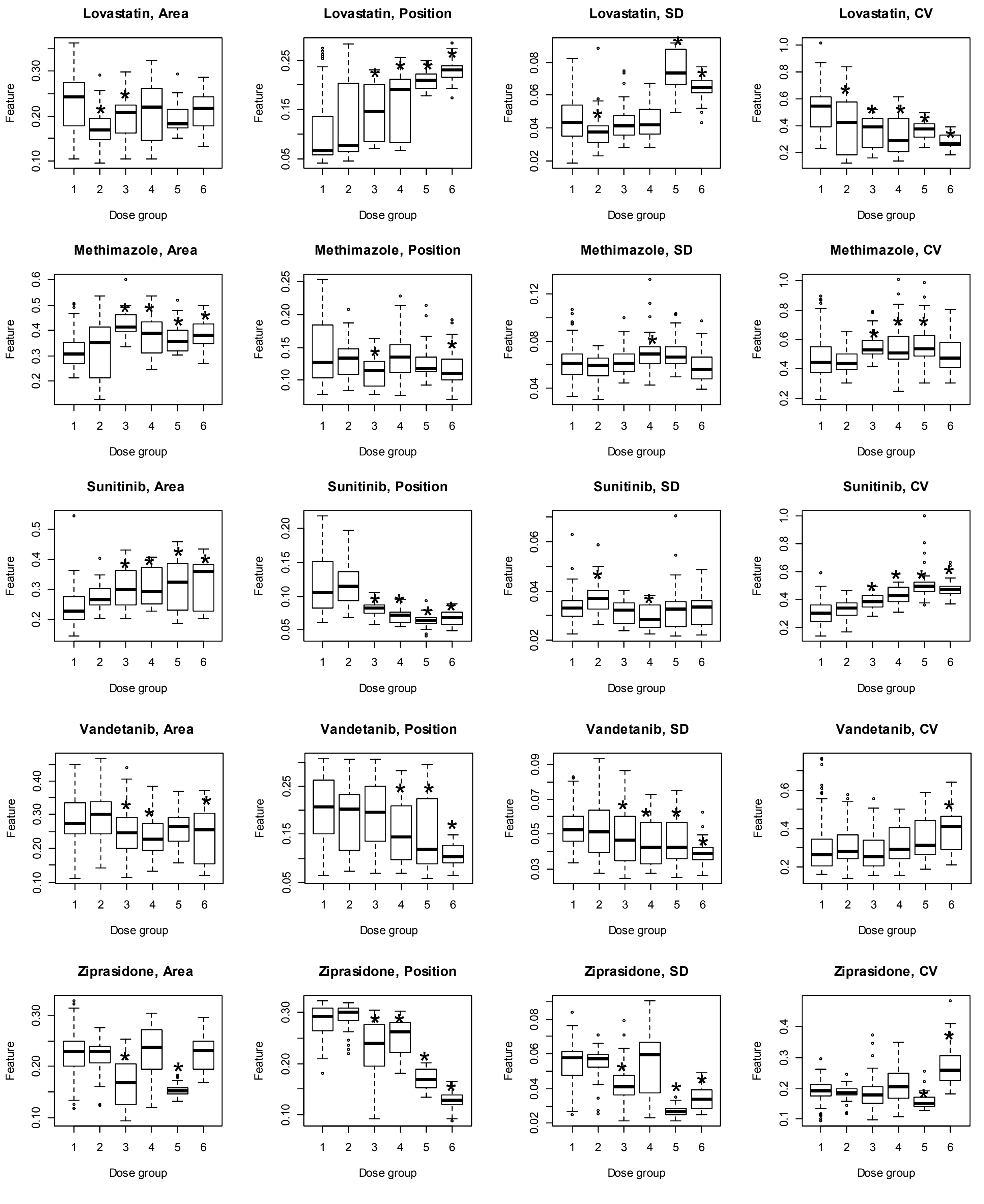

Figure S4 Boxplot of morphologic features for LDC detection (Continued)
Dose groups (1-6): 0, 0.01, 0.1, 0.2, 0.4, 0.8 μg/ml for Lovastatin; 0, 0.3, 1.5, 3, 30,150 μg/ml for Methimazole; 0, 0.03, 0.15,0.18, 0.54, 0.9 μg/ml for Sunitinib; 0, 0.2, 0.3, 0.4, 0.5, 1 μg/ml for Vandetanib; 0, 0.2, 1.25, 2.5, 5, 15 μg/ml for Ziprasidone.
*: $p<0.01$.

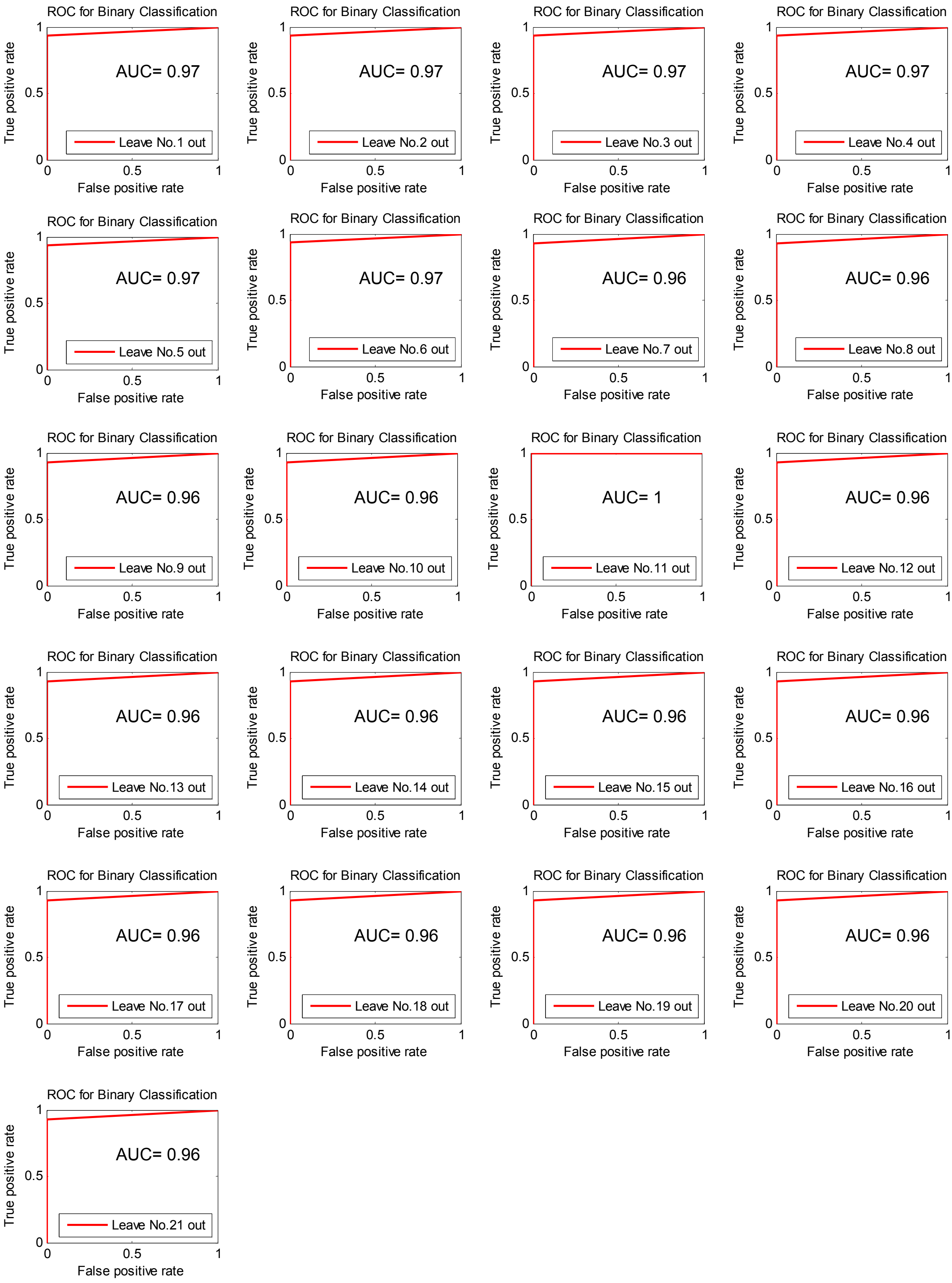

Figure S5 Best receiver operating characteristic (ROC) curves generated from linear comparison of LDC and C_{max} using leave-one-out rule. The corresponding range for the scale factor of C_{max} was 7-10 for all test runs except for Run Leave No. 20 Out, which was 5-10. AUC: area under the receiver operating characteristic curve .

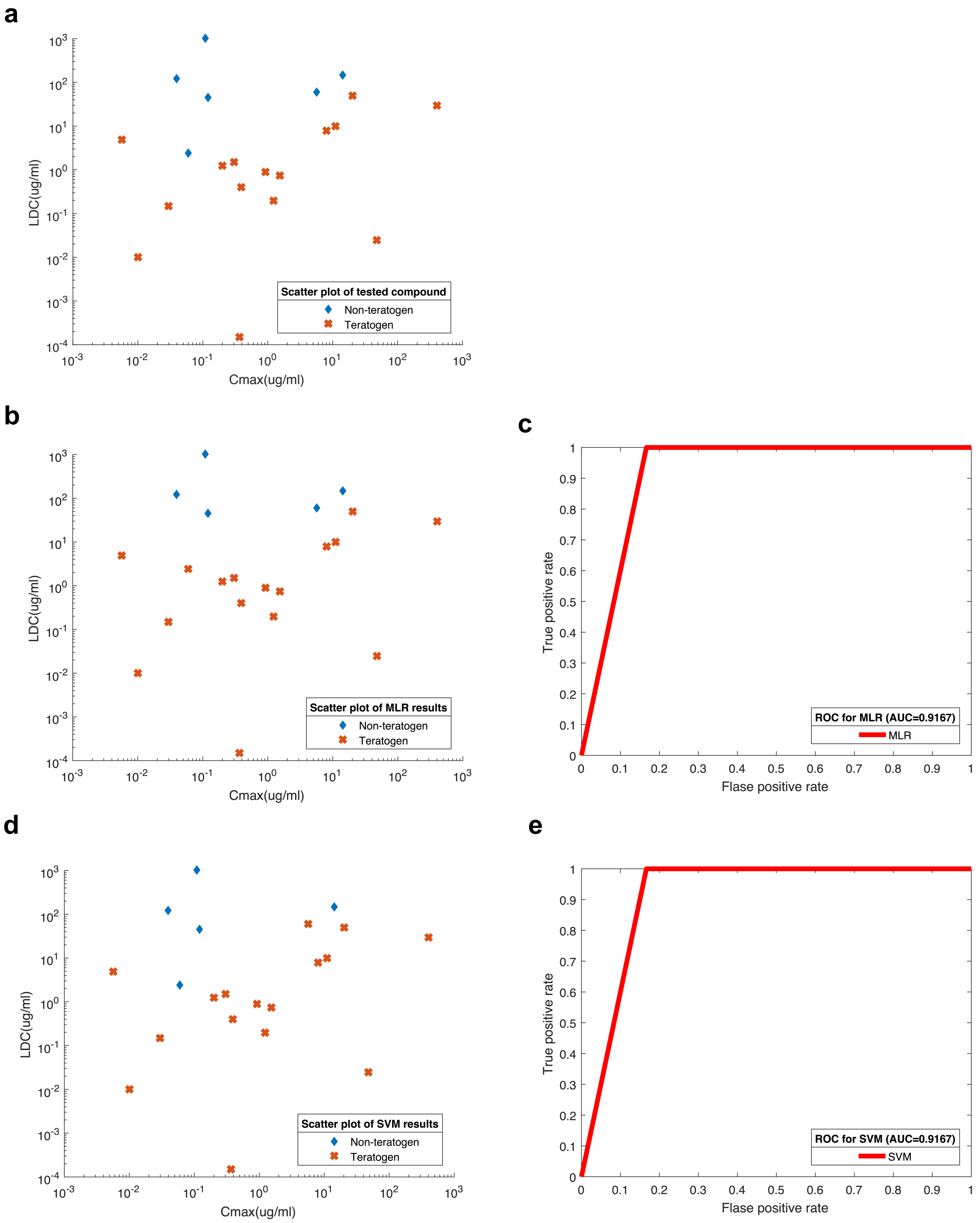

Figure S6 Verification of linear comparison results using MLR and SVM binary classification. (a) Scatter plot of test compounds. (b) Compound classification result using MLR. (c) ROC curve generated from MLR. (d) Compound classification result using SVM. (e) ROC curve generated from SVM.

Table S1 The C_{max} values and references of test compounds. U.S. FDA: U.S. Food and Drug Administration, EMA: European Medicines Agency, Medsafe: New Zealand Medicines and Medical Devices Safety Authority.

Drug name	C_{max} (μg/ml)	Reference
Acetaminophen	24	U.S. FDA Website. http://www.fda.gov/ohrms/dockets/ac/02/briefing/3882b1_13_mcneil-acetaminophen.htm. Accessed 22 February 2017
Acyclovir	5.65	U.S. FDA Website: http://www.accessdata.fda.gov/drugsatfda_docs/label/2008/020487s014lbl.pdf. Accessed 22 February 2017
Amoxicillin	14.4	EMA website. http://www.ema.europa.eu/docs/en_GB/document_library/Referrals_document/Augmentin_30/WC500090608.pdf. Accessed 22 February 2017
Ascorbic acid	156	Padayatty, S. J., Sun, H., Wang, Y., Riordan, H. D., Hewitt, S. M., Katz, A., ... & Levine, M. (2004). Vitamin C pharmacokinetics: implications for oral and intravenous use. Annals of Internal Medicine, 140(7), 533-537.
Caffeine	10	EMA website. http://www.ema.europa.eu/docs/en_GB/document_library/EPAR_-_Product_Information/human/001014/WC500040552.pdf. Accessed 22 February 2017
Diphenhydramine	0.06	EMA website. http://www.ema.europa.eu/docs/en_GB/document_library/Application_withdrawal_assessment_report/2010/04/WC500089539.pdf. Accessed 22 February 2017
Doxylamine	0.12	Videla, S., Cebrecos, J., Lahjou, M., Wagner, F., Guibord, P., Xu, Z., ... & Sans, A. (2013). Pharmacokinetic Dose Proportionality Between Two Strengths (12.5 mg and 25 mg) of Doxylamine Hydrogen Succinate Film-Coated Tablets in Fasting State: A Single-Dose, Randomized, Two-Period Crossover Study in Healthy Volunteers. Drugs in R&d, 13(2), 129-135.
Esomeprazole	1.62	U.S. FDA Website. http://www.fda.gov/downloads/advisorycommittees/committeesmeetingmaterials/pediatricadvisorycommittee/ucm214733.pdf. Accessed 22 February 2017
Folic acid	0.27	Nguyen, P., Boskovic, R., Yazdani, P., Kapur, B., Vandenberghe, H., & Koren, G. (2007). Comparing folic acid pharmacokinetics among women of childbearing age: single dose ingestion of 1.1 versus 5 MG folic acid. The Canadian journal of clinical pharmacology= Journal canadien de pharmacologie clinique, 15(2), e314-22.
Isoniazid	3.09	U.S. FDA Website. http://www.accessdata.fda.gov/drugsatfda_docs/label/2013/050705s010lbl.pdf. Accessed 22 February 2017
Loratadine	0.04	U.S. FDA Website. http://www.accessdata.fda.gov/drugsatfda_docs/nda/2000/20-641S007_Loratadine_biopharmr.pdf. Accessed 22 February 2017
Metoclopramide	0.04	U.S. FDA Website. http://www.accessdata.fda.gov/drugsatfda_docs/nda/2005/021793s000_ClinPharmR.pdf. Accessed 22 February 2017
Methyldopa	7.5	Kwan, K. C., Foltz, E. L., Breault, G. O., Baer, J. E., & Totaro, J. A. (1976). Pharmacokinetics of methyldopa in man. Journal of Pharmacology and Experimental Therapeutics, 198(2), 264-277.
Sitagliptin	0.39	U.S. FDA Website. http://www.accessdata.fda.gov/drugsatfda_docs/label/2012/021995s019lbl.pdf. Accessed 22 February 2017
Thiamine	0.11	Smithline, H. A., Donnino, M., & Greenblatt, D. J. (2012). Pharmacokinetics of high-dose oral thiamine hydrochloride in healthy subjects. BMC Pharmacology and Toxicology, 12(1), 4.

Table S1 The C_{max} values and references of test compounds (Continued). U.S. FDA: U.S. Food and Drug Administration, EMA: European Medicines Agency, Medsafe: New Zealand Medicines and Medical Devices Safety Authority.

**Drug name**	**C_{max} (μg/ml)**	**Reference**
5-Fluorouracil	48.4	Bocci, G., Danesi, R., Di Paolo, A., Innocenti, F., Allegrini, G., Falcone, A., ... & Del Tacca, M. (2000). Comparative pharmacokinetic analysis of 5-fluorouracil and its major metabolite 5-fluoro-5, 6-dihydrouracil after conventional and reduced test dose in cancer patients. Clinical cancer research, 6(8), 3032-3037.
Bosentan	8.17	U.S. FDA Website. http://www.accessdata.fda.gov/drugsatfda_docs/nda/2001/21-290_Tracleer_biopharmr_P2.pdf. Accessed 22 February 2017
Busulfan	1.22	U.S. FDA Website. http://www.accessdata.fda.gov/drugsatfda_docs/label/1999/20954lbl.pdf. Accessed 22 February 2017
Carbamazepine	11	U.S. FDA Website. http://www.accessdata.fda.gov/drugsatfda_docs/label/2010/021710s008lbl.pdf. Accessed 22 February 2017
Diethylstilbestrol	0.0056	Zhang, H., Chen, H., Li, X. J., Zhang, Q., Sun, Y. F., Liu, C. J., ... & Ding, Y. H. (2014). Pharmacokinetics and safety profiles of novel diethylstilbestrol orally dissolving film in comparison with diethylstilbestrol capsules in healthy Chinese male subjects. International journal of clinical pharmacology and therapeutics.
Diphenylhydantoin	20	Medsafe website. http://www.medsafe.govt.nz/profs/datasheet/d/Dilantincapsusptab.pdf
Doxorubicin	0.37	Greene, R. F., Collins, J. M., Jenkins, J. F., Speyer, J. L., & Myers, C. E. (1983). Plasma pharmacokinetics of adriamycin and adriamycinol: implications for the design of in vitro experiments and treatment protocols. Cancer research,43(7), 3417-3421.
Furosemide	400	U.S. FDA Website. http://www.accessdata.fda.gov/drugsatfda_docs/label/2011/018579s029lbl.pdf. Accessed 22 February 2017
Gefitinib	0.93	U.S. FDA Website. http://www.accessdata.fda.gov/drugsatfda_docs/nda/2003/21-399_IRESSA_Clinr.pdf. Accessed 22 February 2017
Imatinib	1.56	EMA website. http://www.ema.europa.eu/docs/en_GB/document_library/EPAR_-_Scientific_Discussion/human/000406/WC500022203.pdf. Accessed 22 February 2017
Lovastatin	0.01	U.S. FDA Website. http://www.accessdata.fda.gov/drugsatfda_docs/label/2002/21316lbl.pdf. Accessed 22 February 2017
Methimazole	0.3	Okamura, Y., Shigemasa, C., & Tatsuhara, T. (1986). Pharmacokinetics of methimazole in normal subjects and hyperthyroid patients. *Endocrinologia japonica*, *33*(5), 605-615.
Sunitinib	0.03	EMA website. http://www.helsedirektoratet.no/helse-og-omsorgstjenester/kreft/kreftlegemidler/vurderinger/Documents/emea-assessment-report-sunitinib-oktober-2007.pdf. Accessed 22 February 2017
Vandetanib	0.4	U.S. FDA Website. http://www.accessdata.fda.gov/drugsatfda_docs/nda/2011/022405Orig1s000ClinPharmR.pdf. Accessed 22 February 2017
Ziprasidone	0.2	U.S. FDA Website. http://www.accessdata.fda.gov/drugsatfda_docs/nda/2001/20-825_Geodan_biopharmr_P1.pdf. Accessed 22 February 2017

Table S2 Compounds for linear comparison leave-one-out cross validation method to find the best scale factor of C_{max}

**No.**	**Drug name**	**C_{max} (µg/ml)**	**LDC (µg/ml)**	***In vivo* teratogenicity**
1	Acyclovir	5.65	60	Non-teratogen
2	Amoxicillin	14.4	150	Non-teratogen
3	Diphenhydramine	0.06	2.4	Non-teratogen
4	Doxylamine	0.12	45	Non-teratogen
5	Metoclopramide	0.04	120	Non-teratogen
6	Thiamine	0.11	1000	Non-teratogen
7	5-Fluorouracil	48.4	0.025	Teratogen
8	Bosentan	8.17	8	Teratogen
9	Busulfan	1.22	0.2	Teratogen
10	Carbamazepine	11	10	Teratogen
11	Diethylstilbestrol	0.0056	5	Teratogen
12	Diphenylhydantoin	20	50	Teratogen
13	Doxorubicin	0.37	0.00015	Teratogen
1	Furosemide	400	30	Teratogen
15	Gefitinib	0.93	0.9	Teratogen
16	Imatinib	1.56	0.75	Teratogen
17	Lovastatin	0.01	0.01	Teratogen
18	Methimazole	0.3	1.5	Teratogen
19	Sunitinib	0.03	0.15	Teratogen
20	Vandetanib	0.4	0.3	Teratogen
21	Ziprasidone	0.2	1.25	Teratogen

Table S3 Teratogen detection results using two-step classification rule for compounds tested previously. All compounds and their relevant information could be found in our previous paper[14].

**Drug name**	**C_{max} (µg/ml)**	**LDC (µg/ml)**	**Step1: Is LDC < = $IC_{25\ aHDF}$ & IC_{25H9}?**	**Step 2: Is LDC <=10 C_{max}?**	**Teratogenicity in µP-hPST**	***In vivo* teratogenicity**
Penicillin G	400 µg/ml	1000 µg/ml	N	-	Non-teratogen	Non-teratogen
Thalidomide	10.922 µM	30 µM	Y	Y	Teratogen	Teratogen
Retinoid acid	347 ng/ml	0.36 ng/ml	Y	Y	Teratogen	Teratogen
D-penicillamine	200 µg/ml	200 µg/ml	Y	Y	Teratogen	Teratogen
Valproic acid	0.574 mM	0.1 mM	Y	Y	Teratogen	Teratogen
